# *SUPT4H1-*edited stem cell therapy rescues neuronal dysfunction in a mouse model for Huntington’s disease

**DOI:** 10.1038/s41536-021-00198-0

**Published:** 2022-01-19

**Authors:** Hyun Jung Park, Areum Han, Ji Yeon Kim, Jiwoo Choi, Hee Sook Bae, Gyu-bon Cho, Hyejung Shin, Eun ji Shin, Kang-in Lee, Seokjoong Kim, Jae Young Lee, Jihwan Song

**Affiliations:** 1 Department of Biomedical Science, CHA Stem Cell Institute, CHA University, 335 Pangyo-ro, Bundang-gu, Seongnam-si, Gyeonggi-do 13488 Korea; 2 Toolgen Inc., 219 Gasan Digital 1-ro, Geumcheon-gu, Seoul, 08594 Korea; 3 iPS Bio, Inc., 3F, 16 Daewangpangyo-ro 712 Beon-gil, Bundang-gu, Seongnam-si, Gyeonggi-do 13522 Korea

**Keywords:** Induced pluripotent stem cells, Development of the nervous system

## Abstract

Huntington’s disease (HD) is a severe inherited neurological disorder caused by a CAG repeat expansion in the huntingtin gene (*HTT*), leading to the accumulation of mutant huntingtin with polyglutamine repeats. Despite its severity, there is no cure for this debilitating disease. HTT lowering strategies, including antisense oligonucleotides (ASO) showed promising results very recently. Attempts to develop stem cell-based therapeutics have shown efficacy in preclinical HD models. Using an HD patient’s autologous cells, which have genetic defects, may hamper therapeutic efficacy due to mutant HTT. Pretreating these cells to reduce mutant HTT expression and transcription may improve the transplanted cells’ therapeutic efficacy. To investigate this, we targeted the *SUPT4H1* gene that selectively supports the transcription of long trinucleotide repeats. Transplanting *SUPT4H1*-edited HD-induced pluripotent stem cell-derived neural precursor cells (iPSC-NPCs) into the YAC128 HD transgenic mouse model improved motor function compared to unedited HD iPSC-NPCs. Immunohistochemical analysis revealed reduced mutant HTT expression without compensating wild-type HTT expression. Further, *SUPT4H1* editing increased neuronal and decreased reactive astrocyte differentiation in HD iPSC-NPCs compared to the unedited HD iPSC-NPCs. This suggests that ex vivo editing of *SUPT4H1* can reduce mutant HTT expression and provide a therapeutic gene editing strategy for autologous stem cell transplantation in HD.

## Introduction

Huntington’s disease (HD) is a progressive neurodegenerative disorder caused by the abnormal expansion of CAG repeats (>40) in the huntingtin gene (*HTT*) exon 1^1^. The expanded CAG trinucleotides encode a polyglutamine stretch that can accumulate into neurotoxic proteinaceous cytoplasmic and intranuclear aggregates^2^. HD patients display progressive brain atrophy with increases in lateral ventricle size. These changes are followed by cognitive deficits, motor control impairment, and psychological symptoms.

There are currently no disease-modifying therapies for HD, consequently resulting in a significant unmet medical need. Effective neurorestorative or neurodegenerative strategies based on human stem cells are potential therapeutics. Previous studies have shown that transplanting human embryo-derived neural stem cells^3^ or mouse-induced pluripotent stem cell (iPSC)-derived neural stem cells^4^ into HD transgenic (TG) mice promoted neuronal or astrocytic differentiation with functional benefits. Patient-specific iPSCs are a potentially renewable source of autologous cells for stem cell therapy that will not induce immune rejection^5–7^. However, iPSCs derived from patients with genetic diseases carry the mutation. Therefore, gene modification before transplantation may increase the therapeutic potential of autologous iPSC therapy.

*HTT* is an evident candidate gene to target in HD because its genetic knockout or knockdown using CRISPR/Cas9, siRNA, or antisense oligonucleotides (ASO) rescues neurotoxicity^8–12^. HTT lowering strategies, including ASO were considered as a promising approach^13,14^. However, these methods will likely knockdown the mutant *HTT* (m*HTT*) and normal *HTT*, thereby ablating the physiological role of normal *HTT*. Normal *HTT* is thought to be required for embryogenesis, since deletion of the HD gene in mice results in early embryonic lethality^15^. Allele-specific targeting of m*HTT* via a single-nucleotide polymorphism (SNP) has been suggested^11,12,16^. However, not all HD patients harbor SNPs to allow for allele-specific knockdown of m*HTT*. Therefore, identifying a universal genetic manipulation to reduce mHTT levels without decreasing wild-type HTT levels would be useful for all HD patients.

SPT4 is a transcription elongation factor that is encoded by *SUPT4H1* and regulates RNA polymerase II processivity^17–19^. One case report indicated that SPT4 is required for specific expression of the trinucleotide and hexanucleotide repeat expansions in HD^20^. Therapeutically targeting SPT4 to specifically reduce the mutant products derived from repeat expansion mutations was investigated. In the case of HD, *SUPT4H1* knockdown reduced mHTT expression in Q81 or Q111 neuronal cells without compensatory increases in wild-type HTT levels. It also decreased mHTT aggregation and toxicity by inhibiting repeated trinucleotides^20^. Furthermore, suppressing the mouse homologue of *SUPT4H1*, *Supt4a*, by administering a *SUPT4H1*-targeting antisense oligonucleotide in the Q175 mouse HD model decreased m*HTT* mRNA and protein expression^21^. Heterozygous deletion of the *Supt4a* gene in R6/2 HD mice also reduced m*HTT* mRNA and protein expression^20^.

For these reasons, we investigated whether targeted ablation of *SUPT4H1* (*SUPT4H1*-edited) in iPSC-NPCs derived from human HD patients increases the therapeutic possibility of autologous stem cell therapy iPSC-NPCs using a mouse HD model. The transplanted *SUPT4H1*-edited HD iPSC-NPCs were better engrafted than the unedited HD iPSC-NPCs at 12 w post-transplantation. Moreover, differentiated neurons and astrocyte were improved similarly. The restoration of healthy neurons and astrocytes consequently increased the striatal intensity of NeuN-positive cells and recovered motor and cognitive functions. These results strongly suggest the potential to develop autologous cell therapy in HD patients through *SUPT4H1*-edited iPSCs that suppress the *HTT* mutation.

## Results

### *SUPT4H1*-edited Q57 HD iPSC-NPCs recovered developmental defects in neurons and astrocytes

To investigate the potential therapeutic effects of *SUPT4H1* knockdown in iPSC-NPCs, we first established NPC differentiation from iPSCs derived from an HD patient with 57 CAG repeats (Cell line ID: ND41656, RUCDR cell line service). We used the in vitro embryoid body-based SFEBq method to differentiate these iPSCs into NPCs.

We screened and identified the optimal region of *SUPT4H1* exon 1 for sgRNA targeting in NPCs using CRISPR/Cas9 (Supplementary Fig. 1a). The sgRNA-targeting noncoding region of the *AAVS1* locus served as a control. Targeted deep sequencing analyses revealed efficient gene editing in the *AAVS1* and *SUPT4H1* loci reaching up to mean of 93.6 and 95.7% total indel efficiencies (mean of 29.5% in-frame, 64.1% out-of-frame mutation for Non-HD, and 27.9% in-frame, 67.9% out-of-frame mutation for Q57 HD iPSC-NPCs) in Non-HD and Q57 HD iPSC-NPCs subsequently (Supplementary Fig. 1a and Supplementary Table 1). To confirm *SUPT4H1* mRNA knockdown following gene editing, we performed qRT-PCR analysis, which revealed robust knockdown of *SUPT4H1* in both Non-HD and Q57 HD iPSC-NPCS with *SUPT4H1* gene editing (Supplementary Fig. 1b). For NPC characterization, we performed immunostaining using NPC-positive markers (anti-SOX2, NESTIN, MUSASHI) and NPC-negative marker (anti-MAP2). No changes in NPC characteristics were observed after *SUPT4H1* gene editing, judged by immunostaining using NPC markers (Supplementary Fig. 1c). IXMC analysis demonstrated that all treated groups showed over 90% positive staining for NPC markers (Supplementary Fig. 1d). Notably, *SUPT4H1* gene editing did not produce changes in cell viability (Supplementary Fig. 1e). Furthermore, no karyotypic changes were detected after *SUPT4H1* gene editing (Supplementary Fig. 2). To confirm SPT4 protein knockdown following gene editing, we performed immunostaining using anti-SPT4 and the NPC marker, anti-NESTIN. SPT4 expression in Q57 HD iPSC-NPCs increased compared to control NPCs (*AAVS1*- or *SUPT4H1*- edited control iPSC-NPCs). Conversely, *SUPT4H1*-edited Q57 HD iPSC-NPCs showed reduced SPT4 expression. Western blot analysis showed the same results (Supplementary Fig. 1f).

**Fig. 1:**
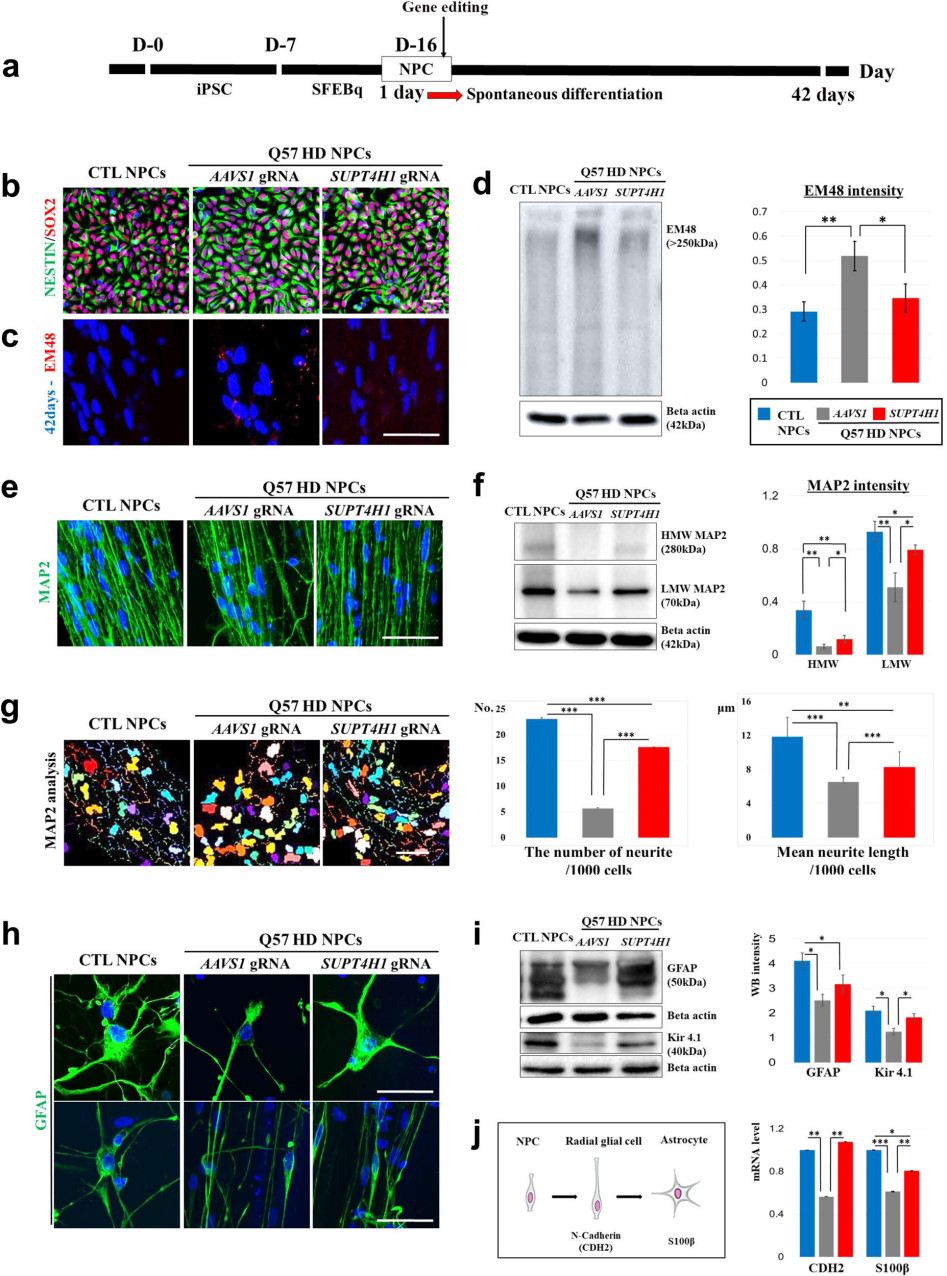
*SUPT4H1* gene editing increased neural differentiation and reduced mHTT protein in Q57 HD iPSC-NPCs. **a** In vitro experimental scheme. The Q57 HD iPSC line was maintained for approximately 7 d (D-7). The dissociated cells were cultured in SFEBq media for 8 d (D-8). Embryoid bodies were dissociated in NPC media, then attempted Neon electroporation. The NPCs were maintained for several days and showed neuronal differentiation after 42 d (after iPSC culture, D-number; after gene editing, number day; blue color). **b** NPC immunostaining (positive marker: NESTIN, SOX2) following gene editing. **c** mHTT protein immunostaining (EM48) demonstrated a decreased HD phenotype in *SUPT4H1*4-edited Q57 HD iPSC-NPCs (*SUPT4H1* gRNA). **d** Western blot analysis of EM48 expression (*n* = 3, *p < 0.05, **p < 0.01). **e** MAP2 immunostaining for morphology exhibited neuronal dendrite maturation in *SUPT4H1*-edited Q57 HD iPSC-derived neurons. **f**. Western blot analysis of HMW and LMW MAP2 expression (*n* = 3, *p < 0.05, **p < 0.01). **g** The number of neurite and mean neurite length were increased in MAP2-positive cells of the *SUPT4H1* gRNA group compared to the *AAVS1*-edited Q57 HD iPSC-derived neuron (*AAVS1* gRNA) (each group: 100 cells, scale bar: 20 μm). **h** GFAP immunostaining. **i** Western blot analysis for GFAP and Kir4.1 revealed normal astrocytic function in the *SUPT4H1*4 gRNA group. (*n* = 3, *p < 0.05, **p < 0.01). **j** Real-time PCR analysis for astrocytic development in AAVS1-edited Q57 HD iPSC-derived NPCs. The CTL (control) and *SUPT4H1*-edited groups showed significant increases in CDH2, a radial glial cell marker, and S100β, a maturate astrocyte marker, mRNA compared to the *AAVS1-*edited group (*n* *=* 3, *p < 0.05, **p < 0.01, and ***p < 0.001). Data were analyzed using two-way ANOVAs followed by Tukey’s post hoc tests with SPSS software or GraphPad Prism. The error bars on the bar charts represent the standard deviation.

Having achieved highly efficient *SUPT4H1* gene editing in HD iPSC-NPCs, we proceeded to spontaneously differentiate them into neural cells over 42 d (Fig. 1a). Before differentiation, we showed NPC characterization using immunofluorescence of NESTIN and SOX2 (Fig. 1b). After differentiation, we performed immunofluorescence and western blotting and found that *SUPT4H1* gene editing reduced EM48 expression, a marker for mHTT protein, compared to *AAVS1* gene editing in HD cells (Fig. 1c, d). To correlate with our western blot data, we performed RT-qPCR and found reduced expression of HTT in *SUPT4H1* gene-edited Q57 HD NPCs (Supplementary Fig. 3).

Interestingly, we observed that *AAVS1*-edited Q57 HD iPSC-derived MAP2- or GFAP-positive cells exhibited different morphology compared to the control or *SUPT4H1-*edited group (Fig. 1e, h). MAP2-positive cell maturation was first identified by western blotting (Fig. 1f). Low molecular weight (LMW) MAP2 isoforms are the immature forms that result from the exclusion of the sequence encoded by exons E7-E9. They are downregulated after the early stages of neuronal development when E7-E9 exon-including high molecular weight (HMW) isoforms are favored^22^. Although HMW-MAP2 expression was observed in the *AAVS1*-edited group, the *SUPT4H1*-edited group showed significantly increased HMW-MAP2 expression compared to the *AAVS1*-edited group.

We next analyzed MAP2-positive cell morphology using MetaXpress for more reliable evidence. We found that the *SUPT4H1*-edited Q57 HD iPSC-derived MAP2-positive cells exhibited significantly more total outgrowths and increased maximum outgrowth length compared to the *AAVS1*-edited Q57 HD iPSC-derived MAP2-positive cells (Fig. 1g). Furthermore, western blotting revealed expression of GFAP and the potassium channel Kir4.1, functional astrocyte markers. The *SUPT4H1*-edited group showed increased GFAP and Kir4.1 expression compared to the *AAVS1*-edited group (Fig. 1i). To identify differentiated neurons and astrocytes, we examined the mRNA expression of GABA and DARPP-32 for mature neurons, and N-cadherin (CDH2) and S100β for radial glial cell and mature astrocytes, respectively. The *SUPT4H1*-edited group showed significant increases in GABA and DARPP-32 mRNA expression, compared to the AAVS1-edited group (Supplementary Fig. 4). In addition, the *SUPT4H1*-edited groups showed significant increases in CDH2 and S100β mRNA compared to the *AAVS1*-edited group (Fig. 1j). Therefore, the *SUPT4H1-*edited Q57 HD iPSC-NPCs with reduced m*HTT* expression underwent normal neural differentiation similar to the control iPSC-derived NPCs.

Although knocking down *HTT* using gene editing reduces mHTT, it also reduces normal wild-type HTT levels, which play a role in neuronal development^15^. To investigate this, we performed *HTT* gene editing using sgRNAs targeting the start codon of the *HTT* gene (Supplementary Fig. 5a). We achieved a similar gene-editing efficiency between *AAVS1*- and *SUPT4H1*-targeting sgRNAs. The *HTT*-edited Q57 HD iPSC-NPCs showed an incomplete differentiation compared to the *SUPT4H1*-edited group. Regarding MAP2-positive cell morphology, the number of total neurites and maximum neurite lengths were significantly reduced in the *HTT*-edited group compared to the control group or *AAVS1*- or *SUPT4H1*-edited groups (Supplementary Fig. 5b, c). Interestingly, the MAP2-positive cells of the *HTT*-edited group did not exhibit increased maturation compared to the *AAVS1*-edited group. As *HTT*-edited Q57 HD iPSC-NPCs showed defective neuronal development, we chose to focus on *SUPT4H1*-edited Q57 HD iPSC-NPCs.

### Functional improvement and neuroprotective effects of *SUPT4H1*-edited Q57 HD iPSC-NPC transplantation in YAC128 HD mice

**Fig. 2:**
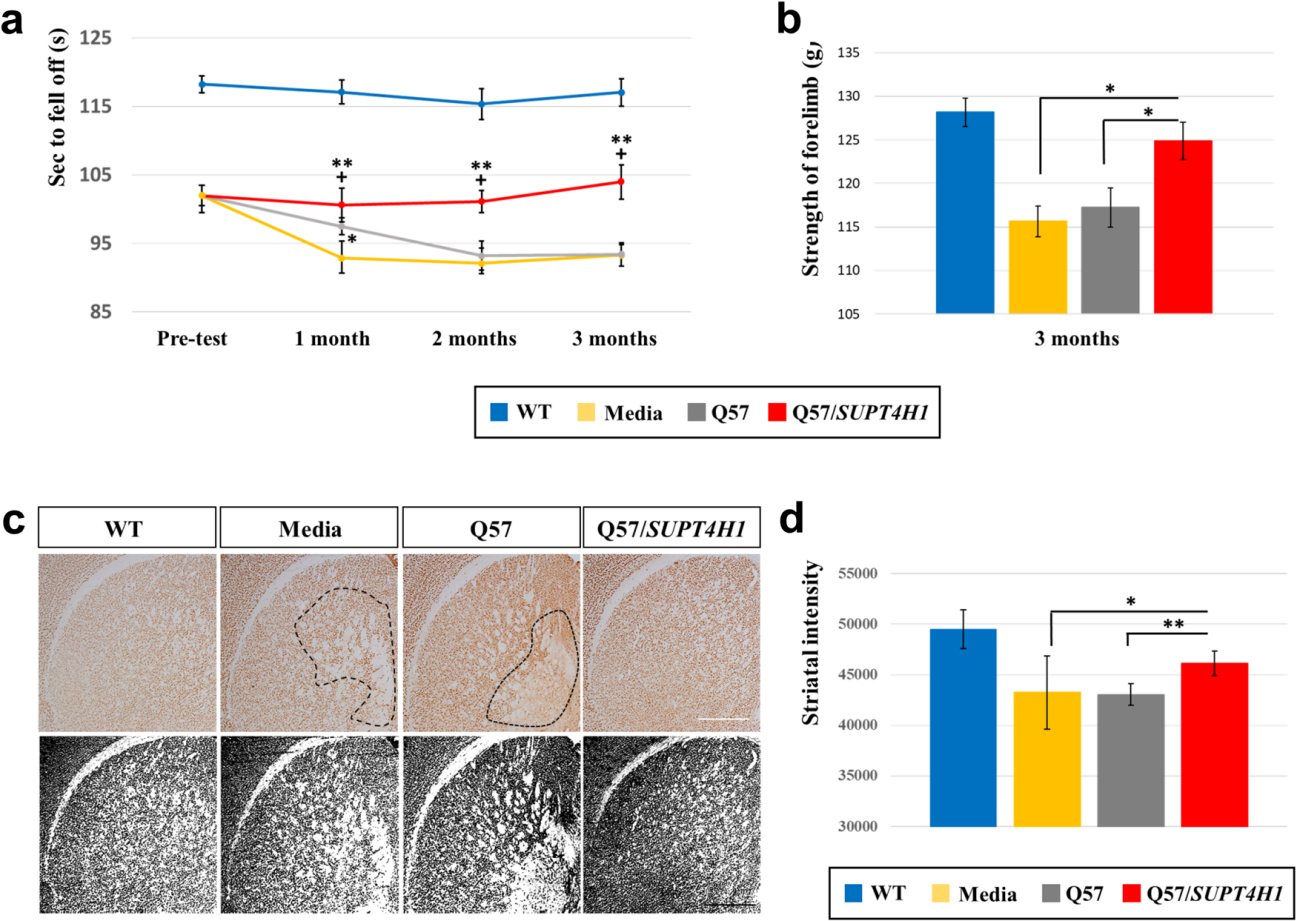
Transplanted *SUPT4H1*-edited Q57 HD iPSC-NPCs promote neuronal regeneration and functional recovery in 9 m-old YAC128 mice. **a, b** Functional motor recovery was monitored every month using the accelerating rotarod and grip strength tests. YAC128 mice transplanted with *SUPT4H1*-edited Q57 HD iPSC-NPCs showed significantly accelerated motor recovery compared to the Q57 HD iPSC-NPCs transplants. All transplant mice significantly recovered at 1 m post-transplantation and this recovery was maintained until 3 m post-transplantation in the *SUPT4H1*-edited Q57 HD iPSC-NPCs group (WT = 9, Media = 8, Q57 = 8, and Q57/*SUPT4H1* = 8; Media vs. Q57 or Q57/*SUPT4H1*: *p < 0.05 and **p < 0.01; Q57 vs. Q57/*SUPT4H1*: ^**+**^*p* < 0.05). **c** Immunostaining revealed an increased NeuN-positive area density in the striatum of YAC128 mice transplanted with *SUPT4H1*-edited Q57 HD iPSC-NPCs (scale bar: 100 μm). **d** Image J analysis showing a significant increase in the striatal density of mice that received *SUPT4H1*-edited Q57 HD iPSC-NPCs transplants (*n* = 3, *p < 0.05, **p < 0.01). Data were analyzed using two-way ANOVAs followed by Tukey’s post hoc tests with SPSS software. The error bars on the bar charts represent the standard deviation.

To compare the therapeutic potential of *SUPT4H1*-edited and unedited Q57 HD iPSC-NPCs, we transplanted these cells into YAC128 mice harboring m*HTT* with 128 CAG repeats. We investigated whether *SUPT4H1*-edited HD iPSC-NPCs rescued motor deficits on the rotarod test in the YAC128 mice at 3 m following transplantation (Fig. 2a). We found that mice injected with unedited Q57 HD iPSC-NPCs showed delays in motor deficits up to 1 m postinjection, whereas mice injected with *SUPT4H1*-edited Q57 HD iPSC-NPCs maintained motor function up to 3 m postinjection. (Fig. 2a; *p* < 0.01). YAC128 mice also typically show deficits in the fine motor control and grip strength. Therefore, we further observed that YAC128 mice transplanted with *SUPT4H1*-edited Q57 HD iPSC-NPCs demonstrated a significantly improved performance at 3 m after transplantation compared to the nonedited Q57 iPSC-NPCs transplanted group (Fig. 2b, *p* < 0.01). These results were comparable to those transplanted with control iPSC-NPCs (Supplementary Fig. 6).

We next investigated whether transplanted *SUPT4H1*-edited Q57 HD iPSC-NPCs (*Q57/SUPT4H1*) provide a protective effect on neuronal survival in the brain of YAC128 mice. Three striatal sections from five mice per group were stained with neuronal nuclei (NeuN) antibody and the intensity of NeuN-positive neurons was quantified using ImageJ software 3 m after media or Q57 HD iPSC-NPC transplantation. The YAC128 mice exhibited significantly reduced NeuN-positive density compared to WT. However, the YAC128 mice transplanted with *SUPT4H1*-edited-Q57 HD iPSC-NPCs showed significantly increased NeuN-positive density (Fig. 2c, d).

### *SUPT4H1*-edited Q57 HD iPSC-NPCs engrafted better than unedited cells in YAC128 mice

**Fig. 3:**
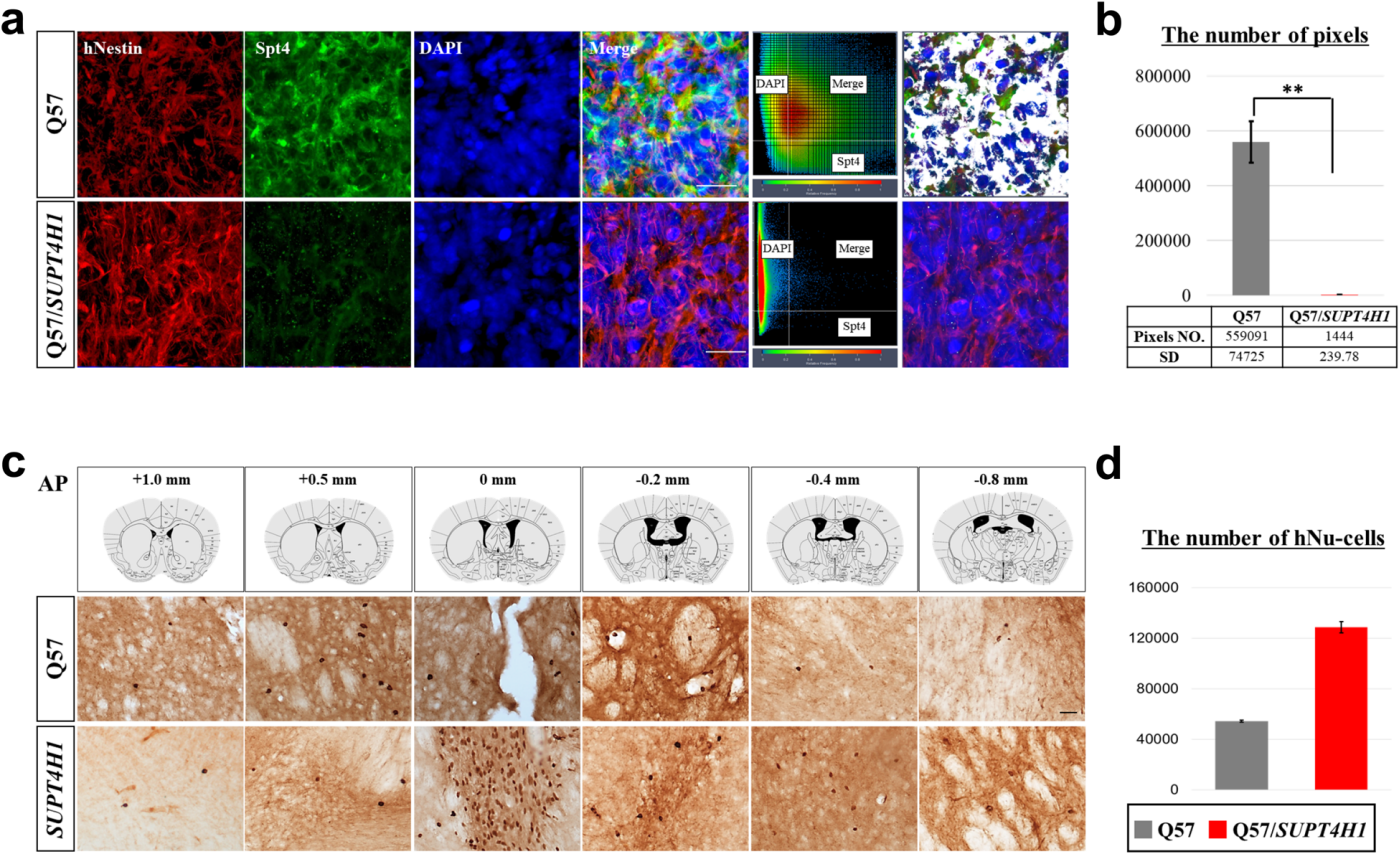
*SUPT4H1*-edited Q57 HD iPSC-NPC transplantation reduced *SUPT4H1* expression and increased cell survival in 9-m-old YAC128 mice. **a** Double staining for transplanted cell markers (hNESTIN) and Spt4 protein revealed that transplanted unedited or *SUPT4H1*-edited Q57 HD iPSC-NPCs decreased Spt4 expression (scale bar: 20 μm). **b** The number of pixels for Spt4 expression quantified using ZEN black software analysis were significantly decreased in transplanted *SUPT4H1*-edited Q57 HD iPSC-NPCs (*n* = 3, **p < 0.01). **c** Immunostaining for hNu 12 week post-transplantation showed human cell survival in the striatum of YAC128 mice transplanted with unedited or *SUPT4H1*-edited Q57 HD iPSC-NPCs (scale bar: 20 μm). **d** Quantification of hNu-positive cell numbers in the 6–7 sections included the striatal lesion site (*n* = 5). Data were analyzed using two-way ANOVAs followed by Tukey’s post hoc tests with SPSS software. The error bars on the bar charts represent standard deviation.

As *SUPT4H1*-edited Q57 HD iPSC-NPCs showed therapeutic efficacy compared to unedited cells, we next investigated the fate of these cells following transplantation. Immunofluorescence analysis for human-specific NESTIN (hNESTIN) confirmed the absence of SPT4 expression in transplanted *SUPT4H1*-edited Q57 HD iPSC-NPCs, indicative of transplanted human cells. However, notable SPT4 expression was found in nonedited cells 1 week following transplantation (Fig. 3a). We counted the pixel numbers of merged hNESTIN and SPT4 in the immunofluorescence images using colocalization analysis of Zen black software (Fig. 3b).

**Fig. 4:**
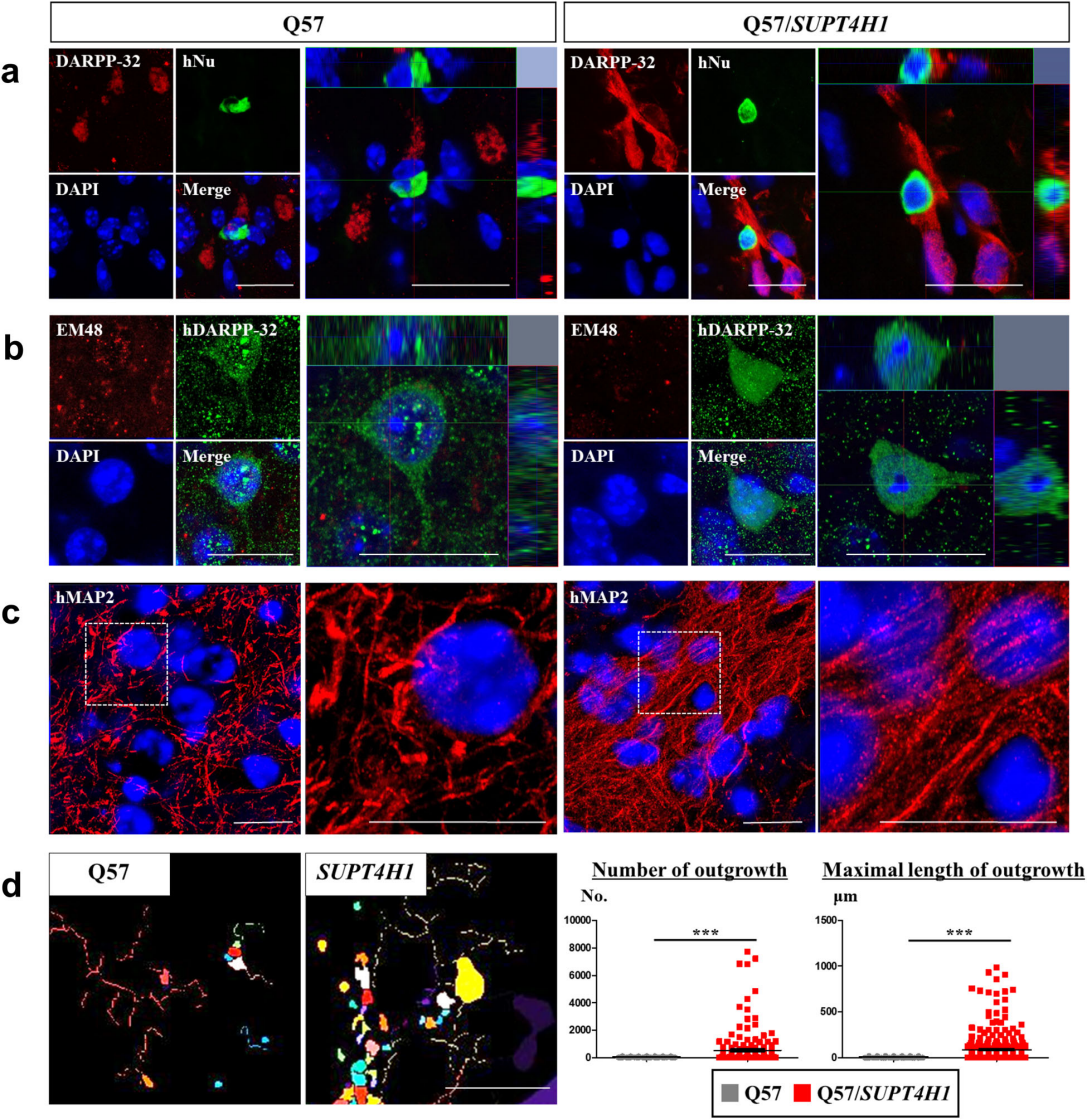
Transplanted unedited or *SUPT4H1*-edited Q57 HD iPSC-NPCs have different neuronal differentiation potential in YAC128 mice. **a** Double staining for the transplanted cell marker (hNu) and MSN marker (DARPP-32) revealed that transplanted unedited or *SUPT4H1*-edited Q57 HD iPSC-NPCs differentiated into MSNs (scale bar: 20 μm). **b** Double staining for the human-specific MSN marker (hDARPP-32) and HD phenotype marker (EM48) in the striatum demonstrated decreased EM48 expression in transplanted *SUPT4H1*-edited Q57 HD iPSC-derived MSNs (scale bar: 20 μm). **c** Immunostaining to identify hMAP2-positive cells exhibited neuronal dendrite degeneration in the transplanted unedited Q57 HD iPSC-NPCs-derived neurons (scale bar: 20 μm). **d** Quantification of neurite numbers and max neurite length indicated more neuronal maturation in transplanted *SUPT4H1*-edited Q57 HD iPSC-NPCs-derived MAP2-positive cells than the unedited cells. (Photograph: same color is one cell for analysis, scale bar: 100 μm, for two regions in 6–7 brain sections of each group, ***p < 0.001). Data were analyzed using two-way ANOVAs followed by Tukey’s post hoc tests or Student’s t tests with GraphPad Prism. The error bars on the bar charts represent the standard deviation.

**Fig. 5:**
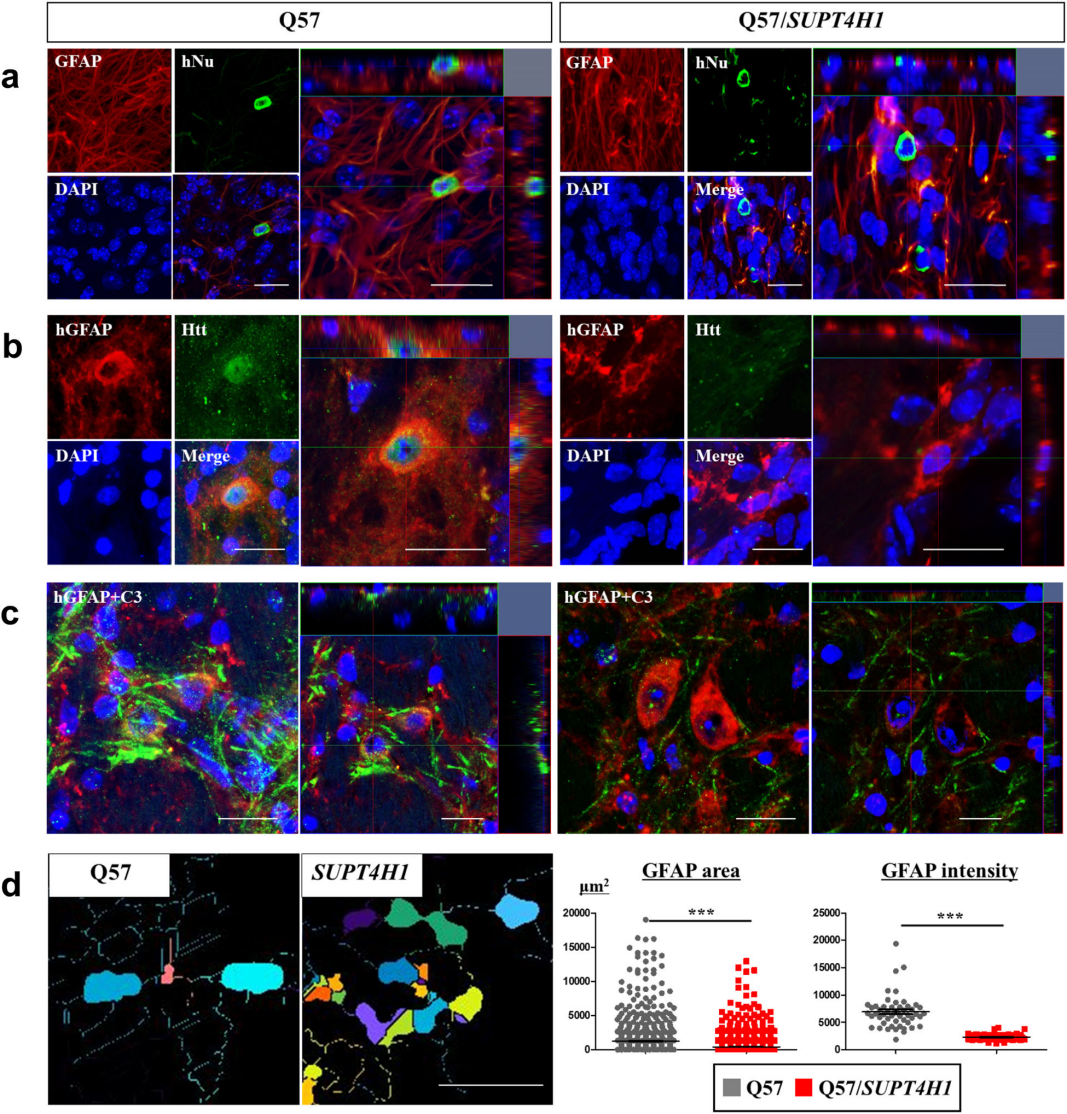
Transplanted unedited or *SUPT4H1-*edited Q57 HD iPSC-NPCs have different astrocytic differentiation potential in YAC128 mice. **a** Double staining for the transplanted cell marker (hNu) and astrocyte marker (GFAP) revealed that transplanted unedited or *SUPT4H1*-edited Q57 HD iPSC-NPCs differentiated into astrocytes (scale bar: 20 μm). **b** Double staining for the human-specific astrocyte marker (hGFAP) and HD phenotype marker (EM48) in the striatum revealed decreased EM48 expression in transplanted *SUPT4H1*-edited Q57 HD iPSC-derived astrocytes (scale bar: 20 μm). **c** Immunostaining to identify C3 expression revealed reactive astrocytes in transplanted unedited Q57 HD iPSC-NPCs-derived astrocytes (scale bar: 20 μm). **d** Quantification of the GFAP-positive area and intensity indicated more activation in the transplanted *SUPT4H1*-edited Q57 HD iPSC-NPCs-derived astrocytes than the unedited cells. (Photograph: same color is one cell for analysis, scale bar: 100 μm, for two regions in 6–7 brain sections of each group, ***p < 0.001). Data were analyzed using two-way ANOVAs followed by Tukey’s post hoc tests or Student’s *t* tests using GraphPad Prism. The error bars on the bar charts represent standard deviation.

Human nuclei (hNu)-positive cells were observed in the striatum of *SUPT4H1*-edited and unedited cell transplantation groups 3 m after transplantation (Fig. 3c), indicating that the transplanted iPSC-NPCs survived in the striatum of YAC128 mice. However, the total number of hNu-positive cells in the striatum of the unedited cell transplantation group was substantially less than in the *SUPT4H1*-edited cell transplantation group (54,280 ± 823 or 128,676 ± 4356 in the striatum, corresponding to approximately 13.5 or 32.2% of the total number of transplanted cells; i.e., 4 × 10^5^) (Fig. 3d). More GFAP-positive cells, co-localized with hNu-positive cells, were observed in YAC128 mice transplanted with the unedited Q57 HD iPSC-NPCs (Fig. 5a) compared to hMAP2-positive cells (Fig. 4c). In contrast, more hMAP2-positive neurons were detected in YAC128 mice transplanted with the *SUPT4H1*-edited Q57 HD iPSC-NPCs (Supplementary Fig. 7).

### Neurons differentiated from SUPT4H1-edited Q57 HD iPSC-NPCs showed improved morphology in YAC128 mice

As more cells survived, we further investigated whether transplanted *SUPT4H1*-edited Q57 HD iPSC-NPCs were likely to differentiate into medium spiny neurons (MSNs) or show other morphology. For this purpose, we double stained slices with hNu and DARPP-32 antibodies (Fig. 4a). The merged hNu/DARPP-32-positive cells in YAC128 mice transplanted with unedited Q57 HD iPSC-NPCs exhibited an unhealthy morphology, such as the defective morphology of DARPP-32-positive neurons, compared to the *SUPT4H1*-edited group (Fig. 4a). The cells in the unedited group had EM48 aggregates that were not present in the *SUPT4H1*-edited group (Fig. 4b). To identify the neuronal maturation morphology, like in the in vitro study, we performed immunofluorescence and MetaXpress software analysis using a human-specific MAP2 (hMAP2) antibody. Interestingly, we found that hMAP2-positive cells in YAC128 mice transplanted with unedited Q57 HD iPSC-NPCs had shorter dendrites and no straight morphology compared to the *SUPT4H1*-edited group (Fig. 4c). The MetaXpress software analysis revealed that the outgrowth numbers and maximum length of hMAP2-positive cells in the *SUPT4H1*-edited group were significantly increased compared to the unedited group (Fig. 4d).

### Astrocytes differentiated from *SUPT4H1*-edited Q57 HD iPSC-NPCs reduced reactive astrocytes in YAC128 mice

We next examined whether transplanted *SUPT4H1*-edited-Q57 HD iPSC-NPCs had the potential to differentiate into astrocytes (GFAP). Slices were double stained with hNu and GFAP antibodies (Fig. 5a). The hNu/GFAP-positive cells in the YAC128 mice transplanted with unedited Q57 HD iPSC-NPCs showed a hypertrophic morphology of reactive astrocytes, compared to the *SUPT4H1*-edited group (Fig. 5a). Furthermore, the cells in the unedited group had HTT aggregates that were not present in the *SUPT4H1*-edited group (Fig. 5b). To identify reactive astrocytes, we double stained slices with antibodies for human GFAP (hGFAP) and complement component 3 (C3), considered a reactive astrocyte marker, and analyzed them using immunofluorescence. We observed increased hGFAP/C3-positive cell expression in YAC128 mice transplanted with unedited Q57 HD iPSC-NPCs compared to the *SUPT4H1*-edited group (Fig. 5c). The MetaXpress software analysis revealed that the area and intensity of GFAP-positive cells in the *AAVS1*-edited group increased significantly and showed reactive astrocyte morphology, but the *SUPT4H1*-edited group did not show reactive astrocyte morphology (Fig. 5d). Therefore, we propose that the YAC128 mouse brain environment, in which mHTT proteins are highly accumulated, converts normal astrocytes into reactive astrocytes.

## Discussion

This study demonstrated that *SUPT4H1*-edited Q57 HD iPSC-NPC transplantation stabilized motor function and improved neuroprotection in the damaged brain of YAC128 mice. We show that *SUPT4H1* gene editing in HD iPSC-NPCs can facilitate normal neural differentiation and functional recovery while avoiding the risks of immunosuppression associated with autologous stem cell therapy. For genetic diseases like HD, ex vivo approaches that combine gene and cell therapy will be highly useful for developing autologous stem cell therapies.

Experimental and preclinical studies supported the commencement of the first neural transplantation clinical trial in HD patients in the 1990s^23^. From human fetal tissue to porcine striatal cells, several cell sources have been used for clinical trials in HD patients^24–28^. However, the results have been highly variable and the clinical benefits were modest for motor^26^ and neuropsychological outcomes^27^. Graft survival and neuronal differentiation have been studied in patients using magnetic resonance imaging^29^. By contrast, iPSCs may produce an unlimited source of patient’s autologous cells that can be used in regenerative medicine^30^. However, mutated genes in autologous cells may cause abnormal neural differentiation and disrupt normal physiological functions, necessitating cell editing for stem cell therapy in HD. In this study, we showed that ablating SPT4 can contribute to the improvement of neurological defects associated with m*HTT* in YAC128 mice.

First, we compared *HTT-* and *SUPT4H1-edited Q57 HD iPSC-NPCs*. m*HTT* causes dysregulation of global gene expression and cellular dysfunction. Therefore, m*HTT* gene knockout or knockdown may be a focus of gene therapy in HD. However, normal and expanded alleles of the endogenous *HTT* gene are not readily distinguished by siRNA or antisense oligonucleotides^11,12^*. HTT* loss and mutation in mouse embryonic stem cells impairs progenitor specification and maturation^31,32^ and targeted genetic modification results in fewer neuronal cells and decreased neurite length^33^. The current study similarly edited the *HTT* gene, which is present in normal and expanded alleles. Our results showed that *HTT*-edited Q57 HD iPSC-NPCs reduced neuronal differentiation and neurite length, indicating that *HTT* gene ablation reduced neural differentiation and the maintenance of neuronal survival in vitro. This might be because our CRISPR/Cas9 *HTT* strategy did not distinguish between the wild-type and mutant alleles. In addition, complete *HTT* knockout might negatively affect neuronal development. Allele-specific targeting of m*HTT* with CRISPR/Cas9 based on SNP associations has recently been developed^16^. However, this approach is likely to be limited by the various associative patterns between SNPs and m*HTT* in patients. In addition, one base pair difference in target sites for CRISPR/Cas9 would likely result in quantitative, but not qualitative, preference in cleavage, increasing the chances of residual cleavage on wild-type *HTT* alleles.

Therefore, we considered that SPT4, a transcription elongation factor, would be the ideal candidate gene for editing HD iPSC-NPCs. Yeast and animal cells with defective SPT4 show reduced synthesis of proteins containing long polyQ stretches, such as mHTT^17,20^. In contrast, *SPT4* gene defects do not alter cellular mRNA synthesis. Furthermore, HD Q175 mice injected with *SPT4* antisense 2′-O-methoxyethyl oligonucleotide and *SPT4*-one-copy-deleted R6/2 mice showed selective decreases in m*HTT* mRNA and protein expression. Furthermore, *SPT4*-one-copy-deleted R6/2 mice showed a prolonged lifespan and delayed motor impairments^17^.

For these reasons, we hypothesized that *SPT4* knockout or knockdown would be the best approach to selectively reduce mHTT. The present study investigated the in vivo and in vitro functions of *SUPT4H14*-edited Q57 HD iPSC-NPCs. Our first result demonstrated that *SUPT4H1*-edited Q57 HD iPSC-NPCs showed decreased SPT4 protein levels. Compared to dual *HTT*-edited Q57 HD iPSC-NPCs, the *SUPT4H1*-edited cells were more likely to differentiate into neuronal cells with increased neurite length and decreased expression of mHTT protein.

Second, our results showed that the unedited Q57 HD iPSC-NPCs were more likely to differentiate into astrocytes than neurons. It has been reported that HD Q140 knock-in NSCs or human HD iPSC (60, 109, and 180Q)-NPCs resulted in fewer NPCs, fewer mature neurons, and more astrocytes due to defects in BDNF expression, which modulates neuronal maturation^34^. Our transplantation results demonstrated that unedited Q57 HD iPSC-NPCs were more likely to differentiate into astrocytes, whereas *SUPT4H1*-edited Q57 HD iPSC-NPCs were more likely to differentiate into mature neurons. Therefore, although further study is required, these results suggest that reducing mHTT via *SUPT4H1* gene editing may enhance neuronal differentiation following in vivo transplantation.

Third, we found that the *SUPT4H1*-edited Q57 HD iPSC-NPCs improved astrocytic function, which is as important as neuronal replacement. HD patients’ brains contain reactive astrocytes with thicker processes, a larger soma, and abnormal function that is associated with reduced glutamate transporter^35^. In addition, abnormal astrocytes are implicated in HD pathophysiology^35–37^. Our results demonstrated that the *SUPT4H1*-edited Q57 HD iPSC-NPCs formed less reactive astrocytes due to reduced mHTT expression. Therefore, *SUPT4H1*-edited Q57 HD iPSC-derived astrocytes may improve the HD brain environment.

In addition, we demonstrated interesting iPSC characterization results. Several studies have demonstrated that HD patients’ iPSCs or their derived NPCs function normally without mHTT expression, based on ubiquitin-protein system activity^38–40^. For example, human embryonic stem cells with m*HTT* exhibit the associated pathology approximately 2 m following differentiation^41^. Patient-derived iPSCs from juveniles and adults with HD showed mHTT expression in neurons after 6 m of culture^42^ or transplantation^43^. The ubiquitin-protein system loses its normal function over the course of neural differentiation^40^. In the present study, YAC128 mice transplanted with unedited Q57 HD iPSC-NPCs exhibited motor recovery at 1 m post-transplantation. However, these mice also had m*HTT*, which was associated with reduced neural differentiation. Therefore, our results demonstrate that mHTT in the unedited Q57 HD iPSC-derived neurons or astrocytes of YAC128 mice may hamper iPSC-NPCs from engrafting and differentiating.

Stem cell therapy is an important therapeutic strategy for several diseases. Numerous clinical and non-clinical studies are actively underway to achieve more effective outcomes. Specifically, some research groups are attempting gene editing to develop stem cells that will not undergo immune rejection. A sufficient supply of autologous stem cells could produce the best treatment if they do not suffer from immune rejection. However, patients with gene mutations, such as in HD, should only have their iPSCs transplanted after gene correction or editing. An easier and more convenient “universal” gene editing approach would be to target the regulatory gene instead of the specific gene in question, which would require more laborious procedures, like gene correction. In the case of HD, we suggest *SUPT4H1* gene editing as an appropriate and efficient strategy to develop autologous stem cell treatment. Furthermore, ex vivo approaches based on combining *SUPT4H1* gene editing and cell therapy may warrant further study for autologous stem cell therapy in other repeat expansion mutation-associated diseases, such as C9FTD/ALS^44^.

## Methods

### Lead contact and material availability

Further information and requests for resources and reagents should be directed to and will be fulfilled by the Lead Contact Jihwan Song (jsong5873@gmail.com). This study did not generate new unique reagents.

### iPSC cell lines

The HD patient iPSC line is the Q57 HD iPSC line (Cell line ID: ND41656) purchased from the RUCDR cell line service. The control iPSC line used in this study was CHAi001-A, which was established from the frozen cord blood of a healthy donor using the episomal method^45^.

### Cell culture and NPC induction

The control and Q57 HD iPSC lines were maintained in Stemfit Basic02 media (Ajinomoto, Japan) supplemented with 100 ng/ml of basic fibroblast growth factor (bFGF, Peprotech) and 10 µM Y27632 (ROCK inhibitor, Peprotech) for approximately 7 d before they were treated with TrypLE solution (GIBCO) for 5 min at 37 °C in a CO_2_ incubator. The dissociated cells were cultured in SFEBq media consisting of DMEM/F12 (Invitrogen) supplemented with 1% antimycotic-antibiotics, 1% nonessential amino acids (NEAA, GIBCO), 0.1% beta-MeOH, 20% Knockout^TM^ serum replacement, 10 µM SB431542, 100 nM LDN193189, and 3× ROCK inhibitor at 37 °C in a CO_2_ incubator for neural induction. The cells were maintained in SFEBq media for 8 days. Embryoid bodies were dissociated in NPC media consisting of DMEM/F12 supplemented with 1:100 antimycotic-antibiotics (Welgene), 1:100 NEAA, sodium pyruvate (GIBCO), d-glucose (Sigma-Aldrich), l-glutamine (Welgene), 1:1000 beta-MeOH, 1:50 B-27 (without vitamin A, GIBCO), and 20 ng/ml bFGF in a dish coated with poly-l-ornithine (Sigma-Aldrich) and laminin (Sigma-Aldrich). Accutase (Stem Cell Technologies) was used to split the cells.

### Preparation of sgRNA

sgRNAs were generated by in vitro transcription using T7 polymerase (New England Biolabs) according to the manufacturer’s protocol. sgRNAs used in this study are listed in Table 1.

**Table 1:** sgRNAs used in this study.

Target site	sgRNA #	Target sequence - PAM (5’ to 3’)
**Human** ***SUPT4H1***	sgRNA 1	CGCAGATGCCGCAGGTCCTT-CGG
**Human** ***AAVS1***	sgRNA 1	ATGGAGCCAGAGAGGATCCT-GGG
**Human** ***HTT***	sgRNA 1	GGAGACCGCCATGGCGACCC-TGG
**Human** ***HTT***	sgRNA 2	CAGCTTTTCCAGGGTCGCCA-TGG

### Gene editing of iPSC-NPCs

For gene editing, ribonucleoprotein (RNP) complex-mediated electroporation was performed as previously described^46^. Briefly, a Neon electroporator (Thermo Fisher Scientific) was used to transfect 1 × 10^5^ iPSC-NPCs with RNP complexes comprised of 1 μg of sgRNA and 4 μg of Cas9 protein (ToolGen). For targeted deep sequencing, genomic DNA (gDNA) was collected from cells 72 h after transfection.

### Targeted deep sequencing

Gene-editing efficiency was verified using targeted deep sequencing as previously described^46^. Briefly, the on-target region was PCR amplified from the gDNA extracted from transfected cells using Phusion polymerase (New England Biolabs). The resulting PCR amplicons were then subjected to paired-end deep sequencing using Mi-Seq (Illumina). The deep sequencing data were analyzed using the online Cas-Analyzer tool (www.rgenome.net). Indels in the region 3 bp upstream from the PAM sequence were considered to be Cas9-induced mutations. The primers used in this study are listed in Table 2.

**Table 2:** Primers used in this study.

Target site	Primer-F (5’ to 3’)	Primer-R (5’ to 3’)
***SUPT4H1***	ACGAGCTATTTACTTCCTGC	CCACCTCTGATTCTGAGAC
***HTT***	CCGCTCAGGTTCTGCTTTTA	TGGAAGGACTTGAGGGACTC
***AAVS1***	CAGTGAAACGCACCAGACG	AATCTGCCTAACAGGAGGTG

### Neural differentiation

Unedited, *SUPT4H1*-, or *HTT* dual-edited Q57 HD iPSCs-NPCs were seeded at a density of 2 × 10^4^ cells on poly-L-ornithine and fibronectin double-coated glass (24 × 60 mm). The medium was changed to neural differentiation medium (NDM) on the following day and changed every 2 days for 6 weeks. The NDM was based on a 1:1 mixed medium of DMEM F12: Neurobasal (GIBCO, Waltham, USA) and composed of 1× Glutamax (GIBCO) and 1% B27 supplement without vitamin A (GIBCO). The cells were fixed for immunocytochemistry and western blot 6 w following differentiation.

### Real-time PCR for GFAP development analysis

The gene-edited cells were washed with RNase-free ice-cold PBS, then total RNA was extracted using TRIzol 6 w following differentiation. RT was conducted using Superscript reverse transcription. Total RNA (1 μg) was used as a template for RT reaction. Quantitative RT-PCR was performed using the SYBR^TM^ Green system (Thermo Fisher Scientific). Amplification was monitored and analyzed by measuring SYBR green binding. For each cDNA, a triplicate amplification was carried out using 2.5 μL (10–100 ng) cDNA, 12.5 mL of Universal PCR Master Mix (Thermo Fisher Scientific), 1 μM each of the primers for CDH2 (immature astrocyte) or S100β, and 1 μl of fluorescent probe (Table 3). The data were recorded as cycle threshold on a Step One Plus from Applied Biosystems.

**Table 3:** Primer information for CDH2 and S100β.

	Primer-F (5’ to 3’)	Primer-R (5’ to 3’)
***CDH2***	CCTCCAGAGTTTACTGCCATGAC	GTAGGATCTCCGCCACTGATTC
***S100β***	ATGTCTGAGCTGGAGAAGG	CTCATGTTCAAAGAACTCGTG

### YAC128 mice and transplantation

All experiments were performed in YAC128 mice maintained on a FVB/N background^47^. Mice were bred in the animal facility at CHA Bio Complex (Pangyo, Korea). Experiments were performed with the approval of the Institutional Animal Care and Use Committee (IACUC 200019) of CHA University. Six-month-old mice were used in the transplantation experiments. We stereotaxically injected a total of 10 mice with 1 µl of unedited or *SUPT4H1*-edited Q57 HD iPSC-NPCs (100,000 cells/µl), respectively into two sites of each hemisphere (a total volume of 4 µl in each mice) using the following coordinates: AP+0.5 mm, ML:±1.8 mm, and DV:−3 and −4 mm from bregma. In the sham group (*n* = 10), 1 µl of suspension medium (DMEM) was injected into two sites of each hemisphere using the same coordinates. No brain edema etc. was observed with hNu staining in the transplanted brains (Supplementary Fig. 8). Both cell-transplanted and medium-injected animals were intraperitoneally injected with cyclosporine A (5 mg/kg, Sigma) 3 d before the transplantation and daily for up to 3 m until they were sacrificed.

### Behavioral tests

Rotarod and grip strength tests were performed over 3 months to evaluate the improvement of motor functions by transplanted cells in 6-m-old YAC128 mice.

#### Accelerating rotarod test

We used an accelerating rotarod protocol (San Diego Instruments) to assess motor coordination and gait changes. Accelerations ranged from 0 to 45 rpm over a period of 2 min. Mice were trained for two trials/d for 3 days. Following the training period, mice were tested for 3 consecutive trials in a single day and allowed 1.5 h of rest time between the trials. The rotarod was wiped clean with ethanol between each subject and trial.

#### Grip strength test

Grip strength was used as an additional assessment of motor function^48^. The apparatus (San Diego Instruments) consisted of an adjustable grip (6 cm wide, 0°–45°) connected to a digital gauge. For this measure, the mouse was lifted by the tail so that its forepaws could grasp the grip. The mouse was then gently pulled backward by the tail until the wire was released. The maximal force exerted before the mouse lost its grip was recorded. Each mouse was tested in 9 replicates, and the average of the 3 highest scores was used for subsequent analyses.

### Western blot analysis

Brain tissues were lysed with RIPA buffer (150 mM NaCl, 1% Nonidet P-40, 0.5% deoxycholic acid, 0.1% SDS, and 50 mM Tris-HCl, pH 7.4) containing a protease inhibitor (Roche), sonicated for 1 min, and incubated on ice for 20 min. The supernatant was collected and the protein concentrations were determined using the BCA assay (Thermo Fisher Scientific) after centrifugation at 13,200 *g* for 15 min at 4 °C. Equal amounts of proteins were then loaded on ~8–12% SDS polyacrylamide gels. The separated samples were transferred to a PVDF membrane, incubated with primary antibodies against the target proteins, and subsequently incubated with HRP-conjugated secondary antibodies. The protein bands were visualized by enhanced chemiluminescence (Millipore) using a bioimaging analyzer (Bio-Rad). The relative intensity of each band was measured using ImageJ software (rsb.info.nih.gov, by W. Rasband). The following primary antibodies were used for western blotting: Spt4 (Cell Signaling 1:1000), EM48 (Millipore, 1:500), MAP2 (Abcam, 1:1000), GFAP (DAKO, 1:1000), and Kir4.1 (Millipore, 1:1000). All blots were derived from the same experiment and were processed in parallel. Un-cropped images of all Western blot results are provided (Supplementary Fig. 9).

### Immunohistochemistry

The YAC128 mice used in this experiment were sacrificed 3 m after cell transplantation following completion of the behavioral analyses. All mice were perfused with saline containing heparin at 5 µl/ml then 4% paraformaldehyde solution. Mouse brains were fixed in 4% paraformaldehyde solution overnight and left to soak in 30% sucrose solution for approximately 3 d. Subsequently, the brain tissues were frozen using an OCT compound and sectioned into 30-µm-thick slices using a cryotome (CM3050, Leica, Germany). The cryosectioned brain tissues were stored in a cryoprotective solution (40% glycerol, 40% ethylene glycol, and 20% 0.2 M phosphate buffer solution) at −20 °C. The slices from each mouse were incubated in a blocking buffer (10% donkey serum and 0.3% Triton X-100 in PBS) for 30 min at room temperature. Thereafter, the samples were incubated overnight with primary antibodies at 4 °C, and then incubated with the appropriate fluorescent probe-conjugated secondary antibodies for 1 h at room temperature protected from light. Nuclei were stained with 4,6-diamidino-2-phenylindole (DAPI, Thermo Fisher Scientific) at a 1:5000 dilution. Images were captured using a confocal microscope (LSM880, Zeiss). The specific primary antibodies used in this study included SPT4 (Biorbyt, 1:200), hNESTIN (R&D Systems, 1:200), SOX2 (Millipore, 1:200), EM48 (Millipore, 1:100), MAP2 (Abcam, 1:200), GFAP (DAKO, 1:200), NeuN (Millipore, 1:200), hNu (Millipore, 1:200), DARPP-32 (Cell Signaling, 1:200), hMAP2 (Thermo Fisher Scientific, 1:200), hGFAP (R&D Systems, 1:200), and C3 (Abcam, 1:100).

### Quantification of hNu-positive cells and striatal density

Unbiased stereological estimation of the total number of hNu-, DARPP-32-, and Iba-1 positive cells, as well as double stained GFAP/hNu-positive cells in the striatum was made using a ImageXpress microconfocal high-content imaging system (Molecular device). The sections used for counting covered the entire striatum over 2 mm, generally requiring 6–7 sections in a series. The positive cells were counted across all regions of the 6–7 sections and analyzed using ImageXpress microconfocal high-content imaging system (Molecular Device) connected to the stage that fed the Z-axis distance information to the computer. The striatum was delineated under a 20x objective. Only the DAPI-merged hNu-stained cells that came into focus within the counting volume (depth of 10 μm) were counted by the scoring module in MetaXpress (Molecular Devices). The total number of cells was calculated according to the optical fractionator formula^49^.

We measured the striatal density of NeuN (Millipore, 1:200)-positive areas to investigate the neuroprotective effects of the transplanted cells. NeuN immunostaining in the striatum was estimated using an optical fractionator and unbiased stereology of stained cells. To measure the striatal density, NeuN immunostaining images were captured in 3 sections near the middle of the striatum from a total of 3 mice. The images were delineated under a 4x objective and measured using ImageJ software (rsb.info.nih.gov, by W. Rasband).

### Morphology analysis and number of MAP2- or GFAP-positive cells

Using an in vitro system, the number of neurites and mean neurite length of MAP2-positive cells were measured in 1000 cells from each culture group. An in vivo system was also used to quantify the number of processes, the maximal process length, the outgrowth intensity, and the body area of MAP2- or GFAP-positive cells in five striatal regions from 6–7 sections of brains from each experimental group (*n* = 3). The outgrowth was measured using the outgrowth module of MetaXpress software (Molecular Device).

For ratio analysis of differentiated transplanted cells in the mice, merged human hNu- and MAP2- or GFAP-positive cells were measured in two whole striatal regions from 6–7 sections of brains from each experimental group (*n* = 3). The number of merged positive cells was measured using the cell sorting module of MetaXpress software (Molecular Devices).

### Real-time PCR (TaqMan probe-based)

For TaqMan probe-based qRT-PCR, 100 ng of cDNA was utilized for each reaction using TaqMan Gene expression master mix according to the manufacturer’s protocol (Thermo Fisher Scientific) on Quantstudio 3. The level of each gene expression was calculated using the C_T_ value, and *GAPDH* was used as an endogenous control (Table 4).

**Table 4:** TaqMan probe Accession numbers.

	Primer-F (5’ to 3’)	Primer-R (5’ to 3’)
***SUPT4H1***	TaqMan probe Accession #: Hs01051404_g1	
***HTT***	TaqMan probe Accession #: Hs00918174_m1	
***GAPDH***	TaqMan probe Accession #: Hs02786624_g1	

### NPC characterization analysis

For NPC characterization analysis, merged human hNu- and NESTIN- or SOX2- or MUSASHI-positive cells were measured in 96 well plate (Table 5). The number of merged positive cells was measured using the cell sorting module of MetaXpress software (Molecular Devices).

**Table 5:** Primers used in this study.

	Primer-F (5’ to 3’)	Primer-R (5’ to 3’)
***Sox2***	GCTGCAAAAGAGAACACCAA	CTTCCTGCAAAGCTCCTACC
***MUSASHI***	ACAGCCCAAGATGGTGACTC	CCACGATGTCCTCACTCTCA
**GABA**	AGAGGTTATGCATGGGATGG	GATGATTGATGTGGTGTGG
**DARPP-32**	CCTGAAGGTCATCAGGCAGT	GGTCTTCCACTTGGTCCTCA
**GAPDH**	GTCATACCAGGAAATGAGCT	TGACCACAGTCCATGCCATC

### Cell viability analysis

Upon gene editing, cells were washed with PBS and detached by TrypLE Select (ThermoFisher). Cells were then centrifuged for 5 mins at 1500 rpm. These were then stained with 0.4% Tryphan Blue and viabilities were measured using automated cell counter FACSSCOPE B (Curiosis).

### Karyotype analysis

Karyotyping was performed using a GTG-banding analysis (Korea Research of Animal Chromosomes, Korea).

### Statistical analysis

Data were analyzed using two-way ANOVAs followed by Tukey’s post hoc tests or Student’s *t* tests, using SPSS software (version 10.0; Chicago, IL) or GraphPad Prism (version 5.0; San Diego). Significance was accepted at the 95% probability level. Data are presented as mean ± SEM.

### Reporting summary

Further information on research design is available in the Nature Research Reporting Summary linked to this article.

## Supplementary information

Supplementary Information

Reporting Summary

## Data Availability

The data that support the findings of this study are available from the corresponding author upon reasonable request.
