# Author Correction : *SUPT4H1*-edited stem cell therapy rescues neuronal dysfunction in a mouse model for Huntington’s disease

**DOI:** 10.1038/s41536-022-00219-6

**Published:** 2022-04-21

**Authors:** Hyun Jung Park, Areum Han, Ji Yeon Kim, Jiwoo Choi, Hee Sook Bae, Gyu-bon Cho, Hyejung Shin, Eun ji Shin, Kang-in Lee, Seokjoong Kim, Jae Young Lee, Jihwan Song

**Affiliations:** 1 Department of Biomedical Science, CHA Stem Cell Institute, CHA University, 335 Pangyo-ro, Bundang-gu, Seongnam-si, Gyeonggi-do 13488 Korea; 2 Toolgen Inc., 219 Gasan Digital 1-ro, Geumcheon-gu, Seoul, 08594 Korea; 3 iPS Bio, Inc., 3F, 16 Daewangpangyo-ro 712 Beon-gil, Bundang-gu, Seongnam-si, Gyeonggi-do 13522 Korea

**Keywords:** Induced pluripotent stem cells, Development of the nervous system

Correction to: *npj Regenerative Medicine* 10.1038/s41536-021-00198-0, published online 19 January 2022

In the Acknowledgements section, “This work was supported by Korean Health Technology R&D Project (NRF-2017M3A9B4061407, NRF-4792018R1C1B6008671, 2018M3A9H3020844) from the National Research Foundation of Korea as well as internal funding from iPS Bio, Inc.” should have read “This work was supported by Korean Health Technology R&D Project (NRF-2017M3A9B4061407, NRF-4792018R1C1B6008671, 2018M3A9H3020844) from the National Research Foundation of Korea.” The original article has been corrected.

