# Supplementary material for: SUPT4H1-edited stem cell therapy rescues neuronal dysfunction in a mouse model for Huntington’s disease
Source: NPJ Regen Med. 2022 Jan 19;7:8. doi: 10.1038/s41536-021-00198-0 (PMC8770473; doi:10.1038/s41536-021-00198-0)
Supplement: Supplementary file 1 — Supplementary Information [file 41536_2021_198_MOESM1_ESM.pdf]

Supplementary Information

SUPT4H1-edited stem cell therapy rescues neuronal dysfunction in a mouse model of Huntington's disease

Hyun Jung Park[1*], Areum Han[1], Ji Yeon Kim[1], Jiwoo Choi[1], Hee Sook Bae[2], Gyu-bon Cho[2], Hyejung Shin[2], Eun ji Shin[2], Kang-in Lee[2] Seokjoong Kim[2], Jae Young Lee[2]*, Jihwan Song[1,3]*

[1]Department of Biomedical Science, CHA Stem Cell Institute, CHA University, 335 Pangyo-ro, Bundang-gu, Seongnam-si, Gyeonggi-do 13488, Korea

[2]Toolgen Inc., 219 Gasan Digital 1-ro, Geumcheon-gu, Seoul 08594, Korea

[3]iPS Bio, Inc., Rm 302, 26 Yatap-ro, Bundang-gu, Seongnam-si, Gyeonggi-do 13522, Korea

*Co-correspondences:

Jihwan Song, E-mail: jsong5873@gmail.com

Jae Young Lee, E-mail: jy.lee2@toolgen.com

Hyun Jung Park, E-mail: pphj0105@hanmail.net

Supplementary Information contains:

9 Supplementary Figures

Supplementary Figure 1.

CRISPR/Cas9 *SUPT4H1* editing in Q57 HD iPSC-NPCs was confirmed by SPT4 knockdown, Related to Figure 1.

Supplementary Figure 2.

All iPSC-NPCs performed karyotype analysis, Related to Figure 1.

Supplementary Figure 3.

SUPT4H1-edited Q57 HD iPSC-NPCs show reduced HTT gene expression level, Related to Figure 1.

Supplementary Figure 4.

SUPT4H1-edited Q57 HD iPSC-NPCs show distinct neural differentiation patterns, Related to Figure 1.

Supplementary Figure 5.

CRISPR/Cas9 dual *HTT*-edited Q57 HD iPSC-NPCs show distinct neuronal differentiation, Related to Figure 1.

Supplementary Figure 6.

YAC128 mice Transplanted with CTL iPSC-NPCs were improved motor function, Related to Figure 3.

Supplementary Figure 7.

Transplanted unedited and *SUPT4H1*-edited Q57 HD iPSC-NPCs show distinct neural differentiation patterns, Related to Figure 3.

Supplementary Figure 8.

At 1 week after transplantation, no obvious morphological changes were observed at the transplantation site in all groups, Related to method.

Supplementary Figure 9.

Un-cropped images of all blots. Related to Figure 1.

1 Supplementary Table

Supplementary Table 1.

Representative targeted deep-sequencing reads (5 most frequent indel patterns) from SUPT4H1 CRISPR/Cas9 treated Q57 HD iPSC-NPC, Related to Figure 1.

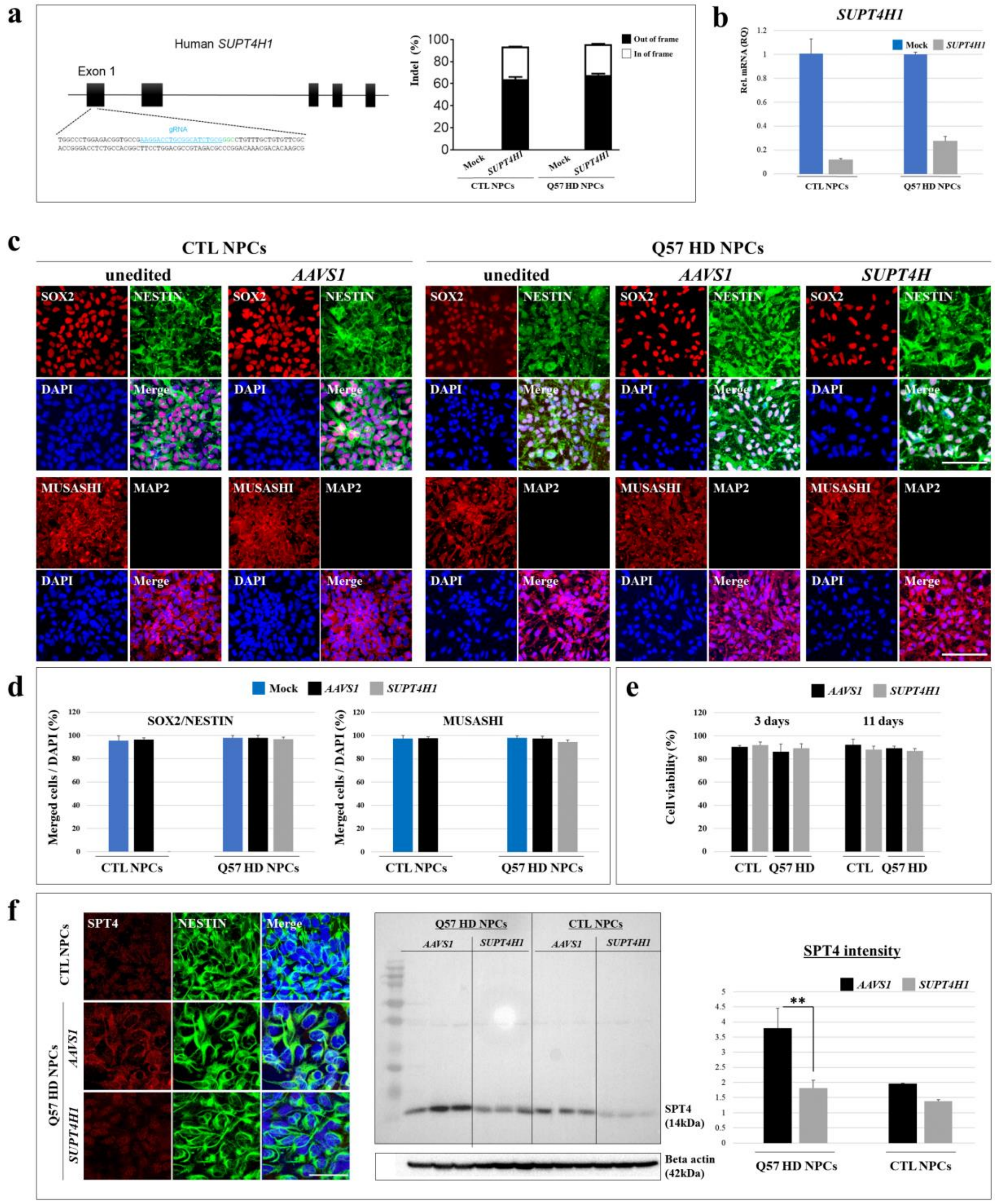

Supplementary Figure 1. CRISPR/Cas9 *SUPT4H1* editing in Q57 HD iPSC-NPCs was confirmed by SPT4 knockdown. **a.** Optical sgRNA targeting of exon 1 of the *SUPT4H1* gene using CRISPR/Ca9 and targeted deep sequencing analysis in *AAVS1* (Control gene)- or *SUPT4H1*-edited HD Q57 iPSC-NPCs. **b.** qRT-PCR analyses of *SUPT4H1*. **c.** Immunofluorescence for SOX2, NESTIN, MUSHASI (positive markers) and MAP2 (negative marker) Scale bar: 100 µm. **d.** All treated groups

showed over 90 % positive staining for NPC markers. **e.** Cell viabilities measured by automated cell counter showed intact viabilities at day 4 and day 11 post electroporation of CRISPR/Cas9 targeting *AAVS1* control or SUPT4H1 in CTL and Q57 HD iPSC-NPCs. **f.** Double staining for NESTIN and SPT4 showed decreased SPT4 in the *SUPT4H1*-edited iPSC-NPCs (Scale bar: 100 μm). Western blot quantification of Spt4 expression showed less Spt4 expression in *SUPT4H1*-edited Q57 HD iPSC-NPCs compared to *AAVS1*-edited cells ($n = 3$, $^{**}p < 0.01$). Data were analyzed using two-way ANOVAs followed by Tukey's post-hoc tests or Student's *t*-tests with GraphPad's Prism. The error bars on the bar charts represent standard deviation.

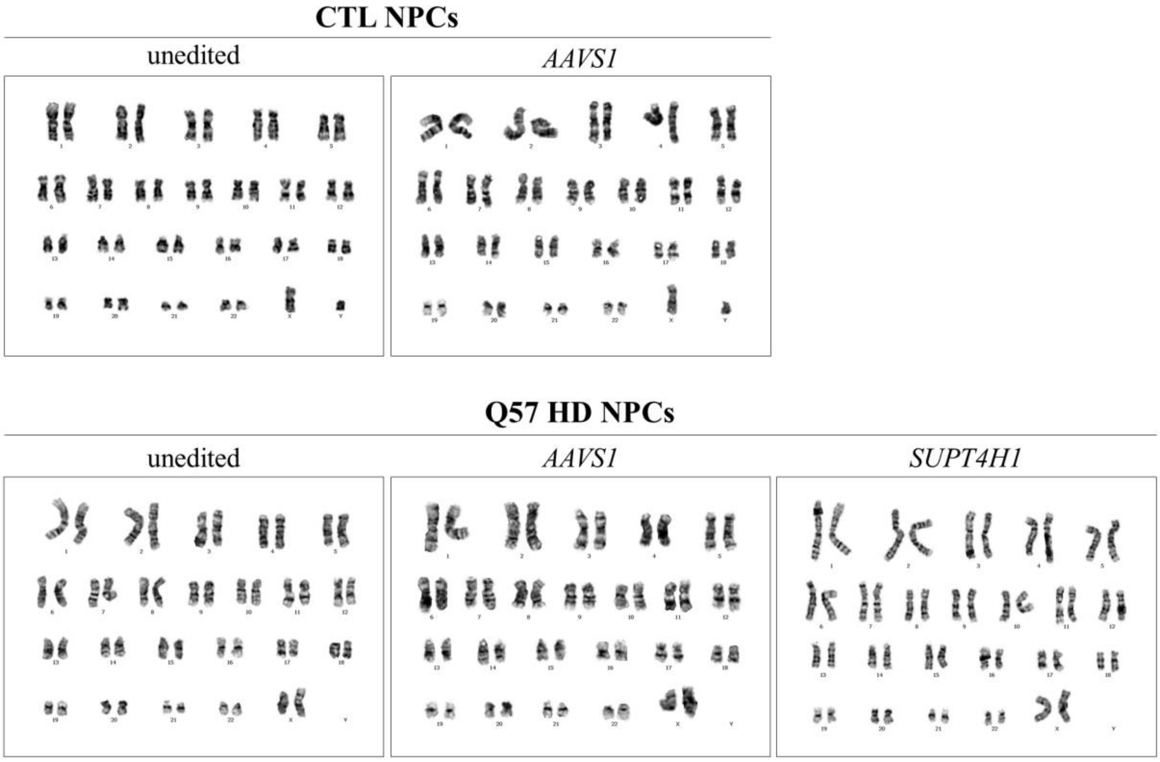

Supplementary Figure 2. Karyotype analyses on all iPSC-NPC groups. No karyotypic changes were detected after *SUPT4H1* gene editing.

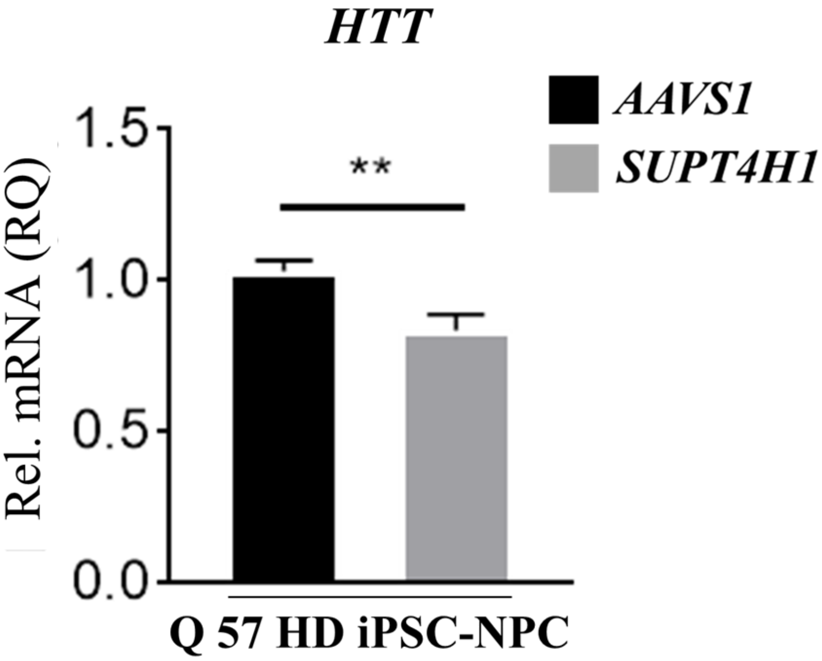

Supplementary Figure 3. *SUPT4H1*-edited Q57 HD iPSC-NPCs showing reduced HTT gene expression level. HTT mRNA expression was examined using qRT-PCR analysis (n=3, **p<0.01). The error bars on the bar charts represent standard deviation.

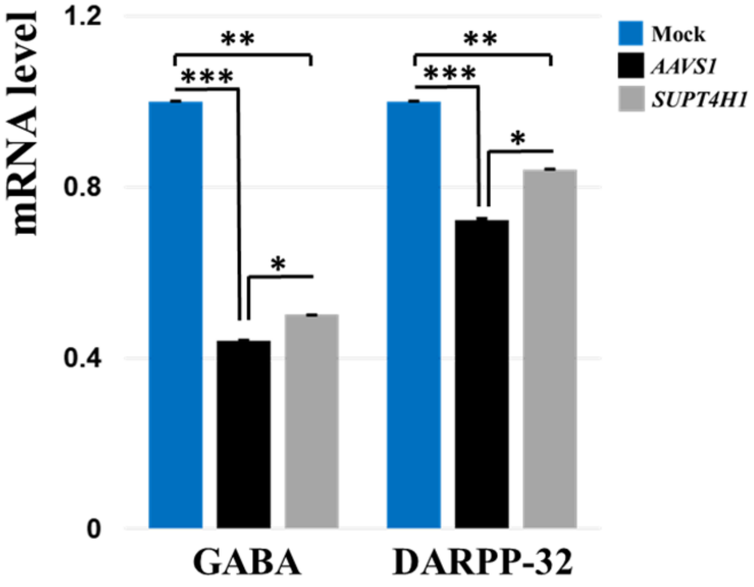

Supplementary Figure 4. *SUPT4H1*-edited Q57 HD iPSC-NPCs showing distinct neural differentiation patterns. mRNA expression of GABA and DARPP-32 for mature neurons are shown (n =3, *p <0.05, ** p < 0.01, and *** p < 0.001). The error bars on the bar charts represent standard deviation.

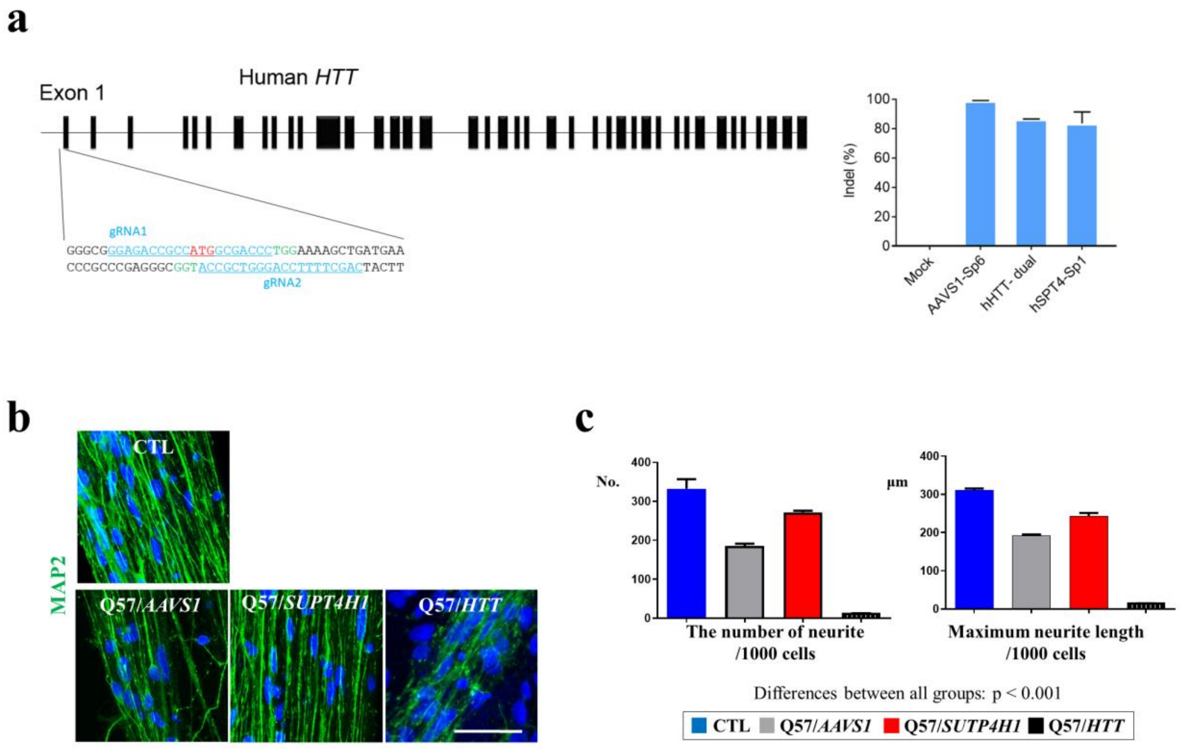

Supplementary Figure 5. CRISPR/Cas9 dual *HTT*-edited Q57 HD iPSC-NPCs showing distinct neuronal differentiation. a. Optical sgRNA targeting of dual *HTT* using CRISPR/Ca9 and targeted deep sequencing analysis in *AAVS1* (Control gene)-, *SUPT4H1*-, or dual *HTT*-edited HD Q57 iPSC-NPCs. **b.** Immunostaining for the neuronal marker MAP2 revealed that dual *HTT*-edited Q57 HD iPSC-NPCs were less likely to differentiate into neurons compared to AAVS1- or *SUPT4H1*-edited cells (scale bar: 20 μm). **c.** Quantification of neurite numbers and mean neurite length of MAP2-positive cells showed less neuronal maturation in dual *HTT*-edited Q57 HD iPSC-derived neurons compared to *AAVS1*- or *SUPT4H1*4-edited cells (n = 1,000 cells, ***$p < 0.001$). Data were analyzed using two-way ANOVAs followed by Tukey's post-hoc tests or Student's *t*-tests with GraphPad's Prism. The error bars on the bar charts represent standard deviation.

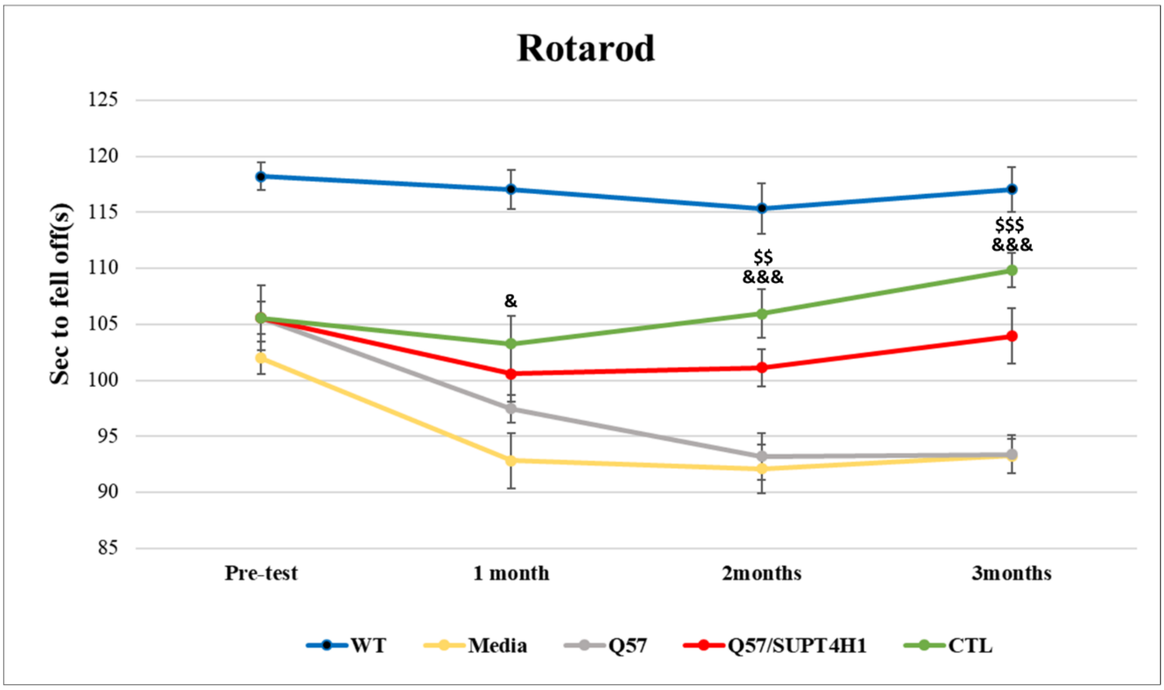

Group	Marker	1 m	2 m	3 m
CTL vs Media	&	$p < 0.05$	$p < 0.01$	$p < 0.001$
CTL vs Q57	$	n.s	$p < 0.001$	$p < 0.001$
CTL vs Q57/*SUPT4H1*		n.s	n.s	n.s

Supplementary Figure 6. YAC128 mice transplanted with CTL iPSC-NPCs showing improved motor function. Functional motor recovery was monitored every month using the accelerating rotarod test. YAC128 mice transplanted with CTL (Control) iPSC-NPCs showed significantly accelerated motor recovery compared to the Q57 HD iPSC-NPCs transplants. However, the CTL group was not significantly different compared to *SUPT4H1*-edited groups (WT = 9, Media = 8, Q57 = 8, and Q57/*SUPT4H1* = 8, CTL = 8). The error bars on the bar charts represent standard deviation.

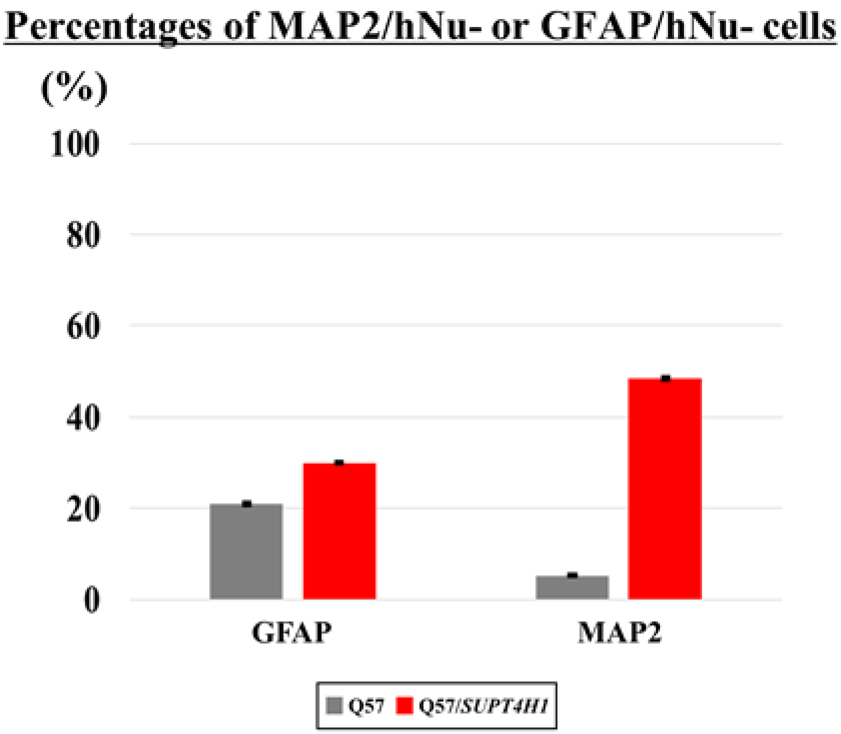

Supplementary Figure 7. Transplanted unedited and *SUPT4H1*-edited Q57 HD iPSC-NPCs showing distinct neural differentiation patterns. Quantification of the number of MAP2- or GFAP-positive cells using the IXMC cell sorting module showed less neuronal differentiation and more astrocytic differentiation in unedited cells compared to *SUPT4H1*-edited cells (n = 5 lesions/section, 6–7 sections/brain from 3 mice, ***$p < 0.001$). Data were analyzed using two-way ANOVAs followed by Tukey post-hoc tests or Student's *t*-tests with GraphPad Prism. The error bars on the bar charts represent standard deviation.

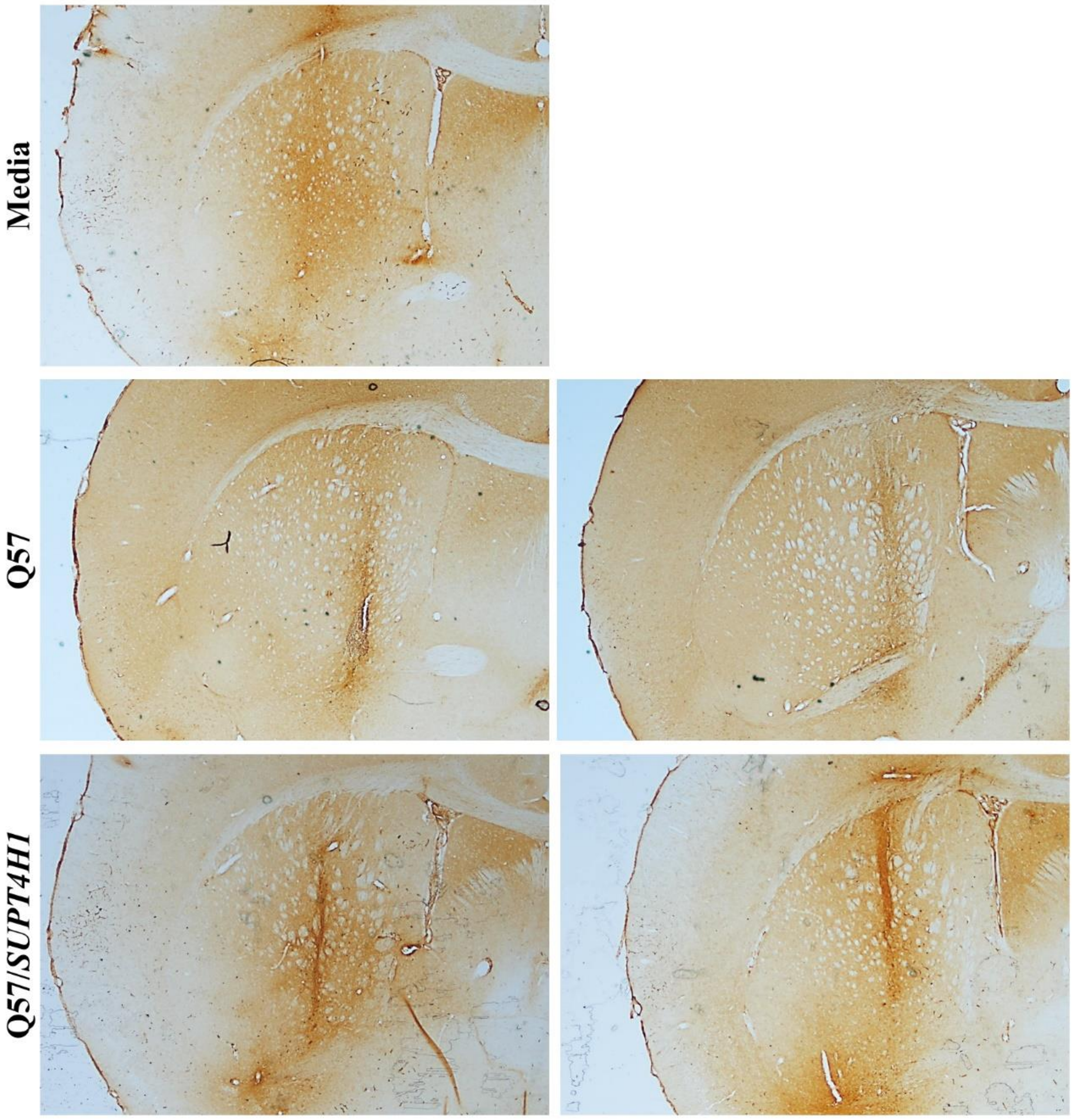

Supplementary Figure 8. At 1 week after transplantation, no obvious morphological changes were observed at the transplantation site in all groups. No brain edema etc. was observed with hNu-staining in the transplanted brains.

Fig. 1d.

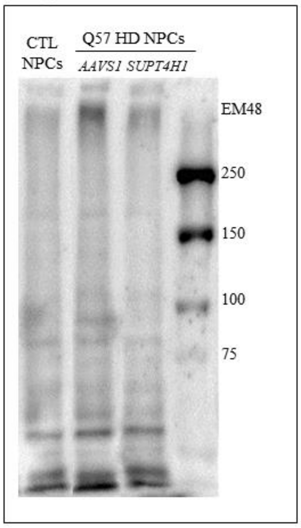

Fig. 1f.

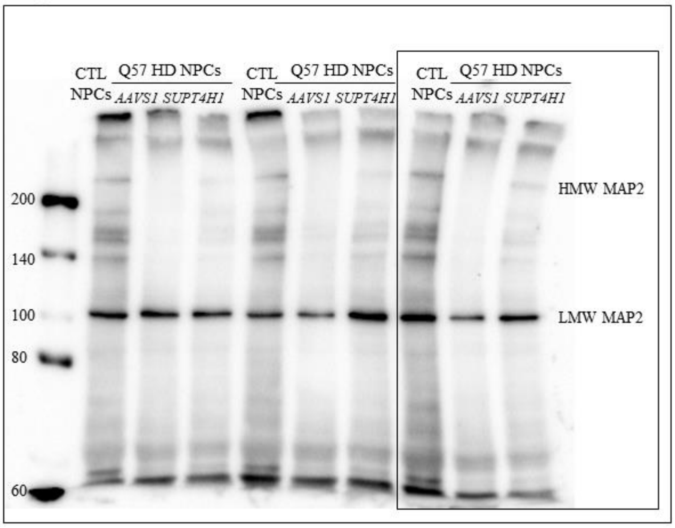

Fig. 1i.

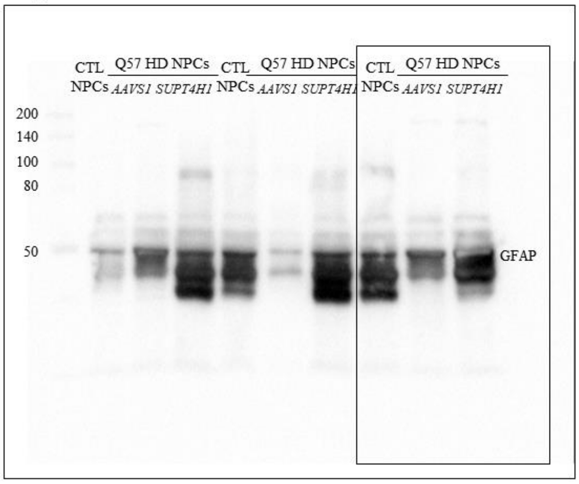

Fig. 1i.

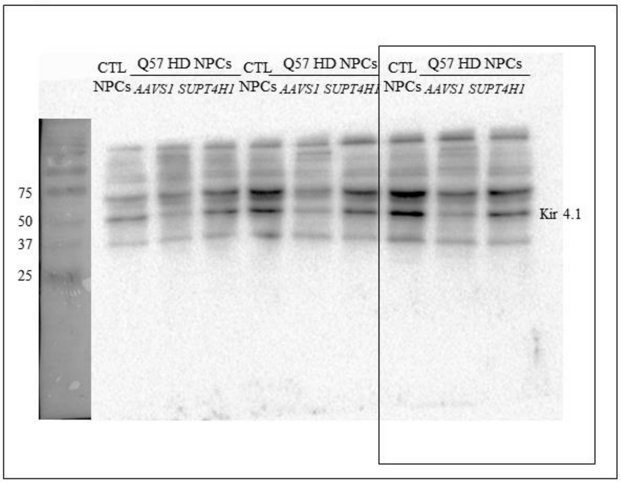

Supplementary Figure 9. Un-cropped images of all Western blot results. Related to Figure 1.

Supplementary Table 1. Representative targeted deep-sequencing reads (5 most frequent indel patterns) from *SUPT4H1* CRISPR/Cas9 treated Q57 HD iPSC-NPC. The PAM sequence in green, and the sgRNA sequence is underlined.

Indel	Local Sequence	In/Out-of-frame
WT	CGGCGAAGATGGCCCTGGAGACGGTGCCGAAGGACCTGCGGCATCTGCGGGCCTGTTTGCTGTGTT	
-1	CGGCGAAGATGGCCCTGGAGACGGTGCCGAA-GACCTGCGGCATCTGCGGGCCTGTTTGCTGTGTT	Out-of-frame
-8	CGGCGAAGATGGCCCTGGAGACGGTG--------CCTGCGGCATCTGCGGGCCTGTTTGCTGTGTT	Out-of-frame
-12	CGGCGAAGATGGCCCTGGAGACGG------------TGCGGCATCTGCGGGCCTGTTTGCTGTGTT	In-frame
-13	CGGCGAAGATGGCCCTGGA-------------GACCTGCGGCATCTGCGGGCCTGTTTGCTGTGTT	Out-of-frame
-8	CGGCGAAGATGGCCCTGGAGACGGTGCCGAA--------GGCATCTGCGGGCCTGTTTGCTGTGTT	Out-of-frame
