# Supplementary material for: SUPT4H1-edited stem cell therapy rescues neuronal dysfunction in a mouse model for Huntington’s disease
Source: NPJ Regen Med. 2022 Jan 19;7:8. doi: 10.1038/s41536-021-00198-0 (PMC8770473; doi:10.1038/s41536-021-00198-0)
Supplement: Supplementary file 2 — Reporting Summary [file 41536_2021_198_MOESM2_ESM.pdf]

nature portfolio

Corresponding author(s): Jihwan Song

Last updated by author(s): Jul 18, 2021

Reporting Summary

Nature Portfolio wishes to improve the reproducibility of the work that we publish. This form provides structure for consistency and transparency in reporting. For further information on Nature Portfolio policies, see our Editorial Policies and the Editorial Policy Checklist.

Statistics

For all statistical analyses, confirm that the following items are present in the figure legend, table legend, main text, or Methods section.

n/a	Confirmed	
☐	☒	The exact sample size (*n*) for each experimental group/condition, given as a discrete number and unit of measurement
☐	☒	A statement on whether measurements were taken from distinct samples or whether the same sample was measured repeatedly
☐	☒	The statistical test(s) used AND whether they are one- or two-sided<br>*Only common tests should be described solely by name; describe more complex techniques in the Methods section.*
☒	☐	A description of all covariates tested
☒	☐	A description of any assumptions or corrections, such as tests of normality and adjustment for multiple comparisons
☒	☐	A full description of the statistical parameters including central tendency (e.g. means) or other basic estimates (e.g. regression coefficient) AND variation (e.g. standard deviation) or associated estimates of uncertainty (e.g. confidence intervals)
☒	☐	For null hypothesis testing, the test statistic (e.g. *F*, *t*, *r*) with confidence intervals, effect sizes, degrees of freedom and *P* value noted<br>*Give P values as exact values whenever suitable.*
☒	☐	For Bayesian analysis, information on the choice of priors and Markov chain Monte Carlo settings
☒	☐	For hierarchical and complex designs, identification of the appropriate level for tests and full reporting of outcomes
☒	☐	Estimates of effect sizes (e.g. Cohen's *d*, Pearson's *r*), indicating how they were calculated

Our web collection on statistics for biologists contains articles on many of the points above.

Software and code

Policy information about availability of computer code

Data collection	No software was used
Data analysis	Data were analyzed using two-way ANOVAs followed by Tukey's post-hoc tests or Student's t-tests, using SPSS software (version 10.0; Chicago, IL) or GraphPad Prism (version 5.0; San Diego). Significance was accepted at the 95% probability level. Data are presented as mean ± SEM.

For manuscripts utilizing custom algorithms or software that are central to the research but not yet described in published literature, software must be made available to editors and reviewers. We strongly encourage code deposition in a community repository (e.g. GitHub). See the Nature Portfolio guidelines for submitting code & software for further information.

Data

Policy information about availability of data

All manuscripts must include a data availability statement. This statement should provide the following information, where applicable:

- Accession codes, unique identifiers, or web links for publicly available datasets
- A description of any restrictions on data availability
- For clinical datasets or third party data, please ensure that the statement adheres to our policy

The data that support the findings of this study are available from the corresponding author upon reasonable request.

Field-specific reporting

Please select the one below that is the best fit for your research. If you are not sure, read the appropriate sections before making your selection.

☒ Life sciences ☐ Behavioural & social sciences ☐ Ecological, evolutionary & environmental sciences

For a reference copy of the document with all sections, see nature.com/documents/nr-reporting-summary-flat.pdf

Life sciences study design

All studies must disclose on these points even when the disclosure is negative.

Sample size	A total of 30 YAC128 mice were used for this transplantation experiment.
Data exclusions	No data from the used animals were excluded.
Replication	No replication of transplantation experiment was performed.
Randomization	Division of animals was randomized.
Blinding	No blinding was applied to this experiment.

Reporting for specific materials, systems and methods

We require information from authors about some types of materials, experimental systems and methods used in many studies. Here, indicate whether each material, system or method listed is relevant to your study. If you are not sure if a list item applies to your research, read the appropriate section before selecting a response.

Materials & experimental systems

n/a	Involved in the study	
☐	☒	Antibodies
☐	☒	Eukaryotic cell lines
☒	☐	Palaeontology and archaeology
☐	☒	Animals and other organisms
☒	☐	Human research participants
☒	☐	Clinical data
☒	☐	Dual use research of concern

Methods

n/a	Involved in the study	
☒	☐	ChIP-seq
☒	☐	Flow cytometry
☒	☐	MRI-based neuroimaging

Antibodies

Antibodies used	SPT4 (Biorbyt, 1:200), hNESTIN (R&D Systems, 1:200), SOX2 (Millipore, 1:200), EM48 (Millipore, 1:100), MAP2 (Abcam, 1:200), GFAP (DAKO, 1:200), NeuN (Millipore, 1:200), hNu (Millipore, 1:200), DARPP-32 (Cell Signaling, 1:200), hMAP2 (Thermo Fisher Scientific, 1:200), hGFAP (R&D Systems, 1:200), and C3 (Abcam, 1:100).
Validation	All antibodies used in this study have been used and validated in the author's laboratory previously.

Eukaryotic cell lines

Policy information about cell lines

Cell line source(s)	The HD patient iPSC line is the Q57 HD iPSC line (Cell line ID: ND41656) purchased from the RUCDR cell line service. The control iPSC line used in this study was CHAi001-A, which was established from the frozen cord blood of a healthy donor using the episomal method (Stem Cells, 2018;36:1552-1566).
Authentication	The CoA document for Q57 HD iPSC line (Cell line ID: ND41656) was provided from the RUCDR cell line service. The control iPSC line, CHAi001-A, was established in the author's laboratory previously (Stem Cells, 2018;36:1552-1566).
Mycoplasma contamination	Mycoplasma contamination was detected using PCR method, which showed no positive results.
Commonly misidentified lines (See ICLAC register)	N/A

Animals and other organisms

Policy information about studies involving animals; ARRIVE guidelines recommended for reporting animal research

Laboratory animals	YAC128 mice were originally purchased from the Jackson Laboratory (FVB-Tg(YAC128)53Hay/J), which were bred in the SPF animal facility at CHA Bio Complex. Both male and female mice were used for transplantation experiment.
Wild animals	N/A
Field-collected samples	N/A
Ethics oversight	Experiments were performed with the approval of the Institutional Animal Care and Use Committee (IACUC 200019) of CHA University.

Note that full information on the approval of the study protocol must also be provided in the manuscript.
